# Learning and Recognition of a Non-conscious Sequence of Events in Human Primary Visual Cortex

**DOI:** 10.1016/j.cub.2016.01.040

**Published:** 2016-03-21

**Authors:** Clive R. Rosenthal, Samantha K. Andrews, Chrystalina A. Antoniades, Christopher Kennard, David Soto

**Affiliations:** 1 Nuffield Department of Clinical Neurosciences, University of Oxford, Oxford OX3 9DU, England, UK; 2 Department of Experimental Psychology, University of Oxford, Oxford OX1 3UD, England, UK; 3 Basque Center on Cognition, Brain and Language, Paseo Mikeletegi, 20009 San Sebastian - Donostia, Spain; 4 Ikerbasque, Basque Foundation for Science, 48013 Bilbao, Spain

**Keywords:** recognition memory, non-conscious memory, primary visual cortex, sequence learning, hippocampus, implicit, relational

## Abstract

Human primary visual cortex (V1) has long been associated with learning simple low-level visual discriminations [1] and is classically considered outside of neural systems that support high-level cognitive behavior in contexts that differ from the original conditions of learning, such as recognition memory [2, 3]. Here, we used a novel fMRI-based dichoptic masking protocol—designed to induce activity in V1, without modulation from visual awareness—to test whether human V1 is implicated in human observers rapidly learning and then later (15–20 min) recognizing a non-conscious and complex (second-order) visuospatial sequence. Learning was associated with a change in V1 activity, as part of a temporo-occipital and basal ganglia network, which is at variance with the cortico-cerebellar network identified in prior studies of “implicit” sequence learning that involved motor responses and visible stimuli (e.g., [4]). Recognition memory was associated with V1 activity, as part of a temporo-occipital network involving the hippocampus, under conditions that were not imputable to mechanisms associated with conscious retrieval. Notably, the V1 responses during learning and recognition separately predicted non-conscious recognition memory, and functional coupling between V1 and the hippocampus was enhanced for old retrieval cues. The results provide a basis for novel hypotheses about the signals that can drive recognition memory, because these data (1) identify human V1 with a memory network that can code complex associative serial visuospatial information and support later non-conscious recognition memory-guided behavior (cf. [5]) and (2) align with mouse models of experience-dependent V1 plasticity in learning and memory [6].

## Highlights

• Sequence learning and recognition memory can operate without visual awareness
• V1, hippocampus, and basal ganglia support learning of a non-conscious sequence
• Old/new status of non-conscious recognition probes modulates V1-hippocampal coupling
• V1 activity predicts non-conscious recognition memory performance

## Teaser

Rosenthal et al. demonstrate that responses in human primary visual cortex (V1) support perceptual learning and recognition of a complex and non-conscious visuospatial sequence, and predict high-level, non-conscious recognition memory performance. These results elaborate on V1’s established role in simple, low-level perceptual learning.

## Results

Recent evidence has identified mouse primary visual cortex (V1) with coding simple serial associations and timing information, when learning a repeating sequence of visible gratings [7], and with a form of recognition memory that is selective for the orientation of visible gratings [6]. In humans, a frontal-occipital negative-going event-related potential (ERP) signal has been identified with a guess response that is associated with the recognition of single visible items [8]. It is thus conceivable that experience-dependent changes in human V1 may be relevant for later recognition memory-guided behavior, but critically, this needs to be established outside of the general modulation in V1 associated with visual awareness [9].

**Figure 1:**
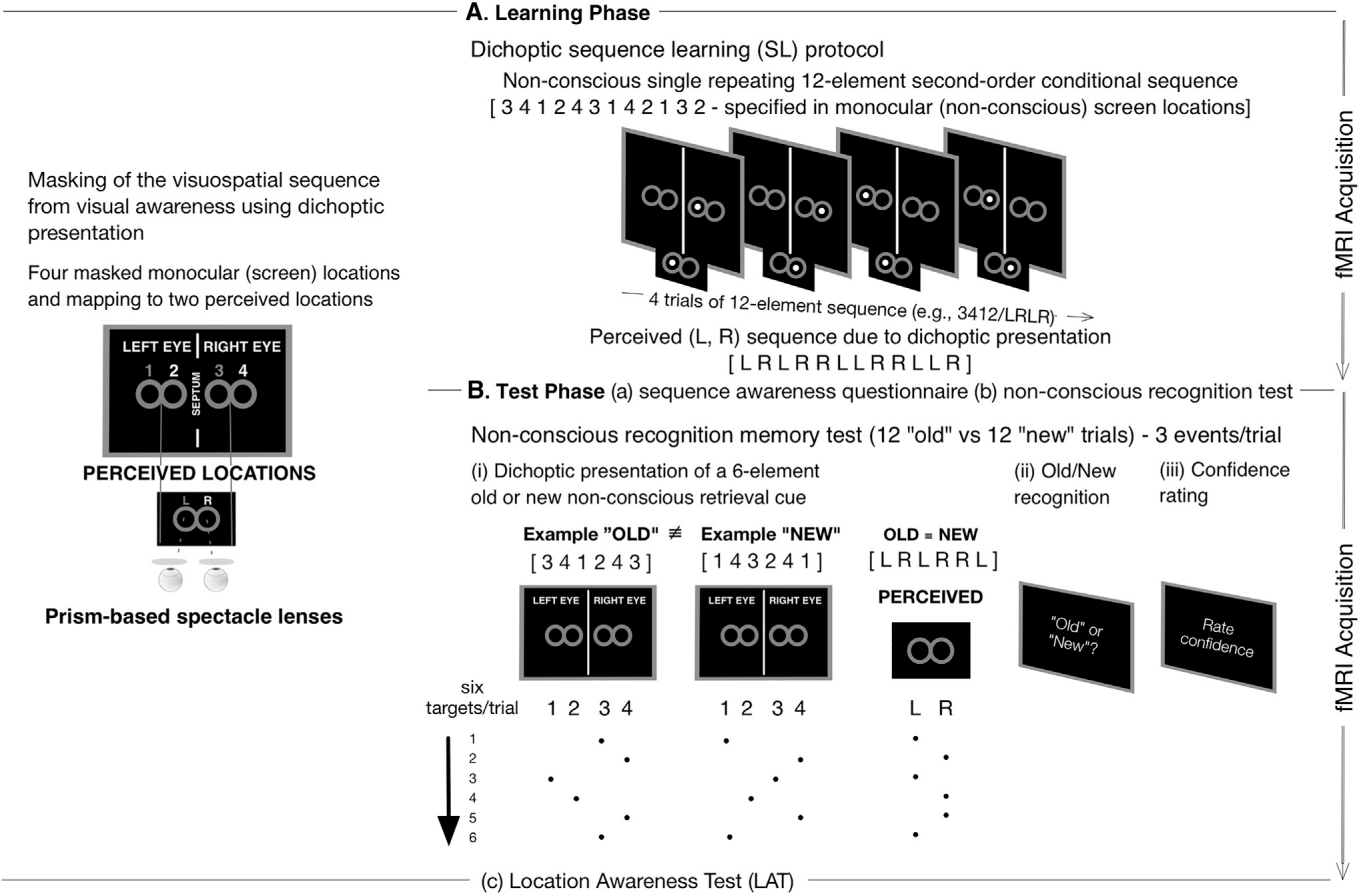
Experimental Procedure and Stimuli (A) Learning phase. First, in a learning phase that lasted approximately 1 hr 15 min, a visuospatial sequence based on a 12-element second-order conditional (SOC) rule specified across four monocular locations (1-2-3-4) was presented repeatedly to induce discontinuous relational coding of serial interocular and monocular associations. Each target was presented for 2000 ms at the center of one of four monocular locations circumscribed by two horizontally aligned and isoluminant figures-of-eight (read from left to right; placeholders 1 and 2 were in the left figure-of-eight and stimulated the left eye whereas placeholders 3 and 4 were in the right figure-of-eight and stimulated the right eye). The four monocular locations were continuously masked from visual awareness by dichoptic presentation: a septum inside the bore of the scanner and spectacles fitted with prism lenses were worn by observers to create two independent visual channels for each eye, which led to the binocular fusion of the four monocular locations into two perceived locations, circumscribed within a single, fused, centrally positioned, and horizontally aligned figure-of-eight. Monocular locations 1 [left eye-of-origin channel] and 3 [right eye-of-origin channel] were perceived in the left placeholder (L), whereas monocular locations 2 [left eye-of-origin channel] and 4 [right eye-of-origin channel] were perceived in the right placeholder (R). This method of dichoptic presentation eliminated crosstalk between each eye-of-origin, enabled binocular fusion to be sustained for long periods, and provided continuous masking of the four monocular target locations in which the second-order conditional sequence was embedded. Observers maintained a fixed head position and were encouraged to attend to the stimuli at the two perceived locations by the requirement to count the number of perceived large-diameter targets across each block of trials. (B) Test phase (three discrete stages). (a) Sequence awareness questionnaire: observers responded immediately after the learning phase to indicate whether they had detected any regularities in the stimulus presentation. (b) Non-conscious recognition memory test (for behavioral results, see Figures S2 and S3 and Tables S1 and S2): sequence knowledge was assessed on the unexpected recognition test (implemented using dichoptic presentation) administered 15–20 min after the learning phase. Each trial of the recognition test (b) involved three phases: (i) observers were presented with a retrieval cue comprised of a six-element sequence of targets (7.2 s), which was drawn from six-element segments of either the old (trained) 12-element SOC sequence or a new (untrained) 12-element SOC sequence. 12 old and 12 new recognition trials were presented in a random order. Notably, the perceived serial order of old and new retrieval cues was equated, as were all other stimulus dimensions and structural properties that would otherwise serve as a basis for a perceived difference that could enable discrimination (i.e., the frequency with which each location occurred, transitions between the four locations, reversals, and laterality) (see also Table S3). (ii) Observers were asked to perform an old/new recognition-based discrimination response during a limited time window of 8 s. (iii) Observers were required to rate their confidence in the old or new response (8 s) on a six-point scale (if “old”/trained, assign a value ranging in confidence between 1 [certain] and 3 [least certain], or, if “new”/untrained, assign a value between 4 [least certain] and 6 [certain]). (c) Location awareness test (LAT) (for results, see Figure S1): the LAT was administered inside the scanner to test the efficacy with which the four monocular locations were masked from visual awareness. Each trial of the LAT presented observers with a target at one of the four monocular positions. Participants were instructed on the mapping between each monocular stimulus and the corresponding perceived binocular location and were then asked to discriminate between monocular locations 1 and 3 for “left” perceived targets or between monocular locations 2 and 4 for “right” perceived targets. Targets remained on screen until a manual response was entered on the response pad and were separated by a 200 ms inter-stimulus interval.

We combined fMRI with a novel dichoptic masking protocol, involving separate learning and recognition phases associated with the presentation of a complex and non-conscious visuospatial sequence of targets appearing across four monocular locations. Figure 1 illustrates the protocol (see Figure 1 legend for details; see also Movie S1 and Supplemental Experimental Procedures for further details).

This visual presentation protocol was expected to be associated with V1 activity because monocular inputs are retained in early visual cortex [10] and dichoptic masking has been identified with responses in V1 [11]. During the learning phase, we ensured that the observers sustained their attention to single targets that appeared on each trial by asking them to count the number of trials with a large-diameter target (LDT; targets could be of either standard or large size—see Supplemental Experimental Procedures). Performance on the LDT counting task was at ceiling (see Supplemental Results). The LDT counting task was also expected to drive V1 activity, because V1 codes for magnitude differences between stimuli [12].

An unexpected non-conscious recognition memory test followed 15–20 min after the learning phase (see Figure 1 and its accompanying legend for a description of the recognition procedure and the features of the retrieval cues). On each trial of the recognition test, observers were asked to discriminate whether the retrieval cue was drawn from the old (trained) sequence or from a new sequence and to rate the confidence in their response on a six-point scale (1–3, “old”; 4–6, “new”; see Figure 1). Crucially, old and new retrieval cues were perceived in the same serial order due to dichoptic presentation. As can be seen in Figure 1, the sequence of locations that distinguished old (e.g., 3-4-1-2-4-3) from new retrieval cues (e.g., 1-4-3-2-4-1) was confined to the monocular level. For the above examples, both old and new retrieval cues were perceived as L-R-L-R-R-L (see Table S3 and Figure 1). Therefore, serial order information that distinguished old and new retrieval cues was not available to visual awareness (as confirmed by psychophysical tests reported below).

Before presenting the neuroimaging data, we report the results from behavioral control experiments demonstrating that (1) the participants did not have conscious access to the four (monocular) locations (see Figure S1 and Supplemental Results) and (2) sequence learning operated independently of motor-based mechanisms, i.e., did not involve somatomotor responses and eye movements that would generate stimulus-motor response bindings coinciding with the structure of the visuospatial sequence (cf. [4, 13]). Observers’ inability to identify the monocular locations was consistent with a lack of knowledge, even about the overall basic sequence structure (as assessed on a post-learning awareness questionnaire; see Supplemental Results).

### Neural Substrates of Sequence Learning

**Figure 2:**
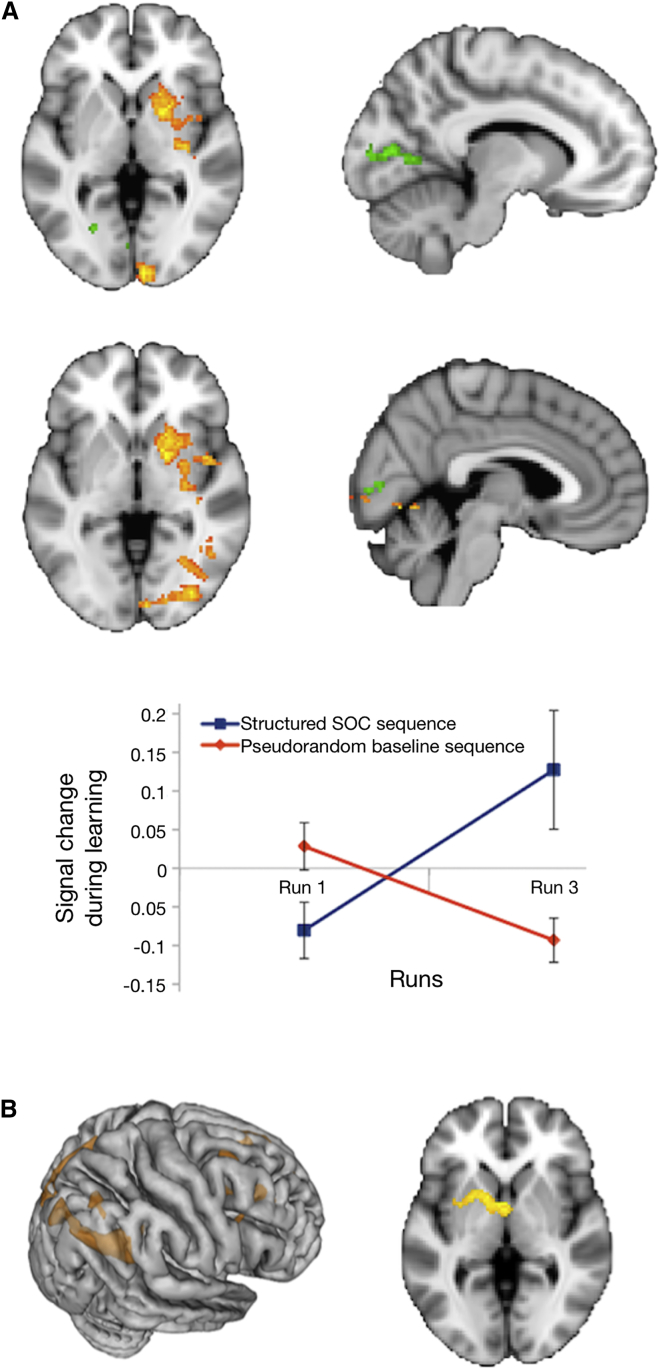
Changes in BOLD Activity Associated with Non-conscious Learning and Learning a Visible Sequence (A) Brain regions exhibiting linear changes in BOLD signal for structured and pseudorandom sequences across the dichoptic learning phase (Z > 2.3, p < 0.05, whole-brain corrected; [14]), rendered onto axial and sagittal views. The yellow clusters depict the brain areas in which the learning effects across runs were higher for the structured sequence blocks relative to the pseudorandom blocks. We computed within-run statistical contrasts that were sensitive to the nature of structured (S) and pseudorandom (R) sequences, namely, for the repetition of the structured sequence (i.e., S3 > S4: [S1-S2-R1-S3-R2-S4]) on each of the three training runs. Correspondingly, for each run of the learning phase, a contrast test for the attenuation of the neural response with the repetition of structured sequences (e.g., S3 > S4) was derived (and likewise for the pseudorandom sequences, e.g., R1 > R2). The respective within-run estimates for the structured and for the pseudorandom sequences were submitted separately to across-run within-subject fixed-effects analyses, testing for linear modulations across the three training runs. Finally, we performed a group-level paired t test to assess which brain regions were associated with increased training effects in the structured relative to the pseudorandom sequence (and vice versa). The graph of signal change during learning depicts the linear estimates of these neural repetition effects across fMRI runs (average of all clusters of activity depicted in yellow; error bars correspond to SEM). Hence, the linear increase for structured blocks (in blue) reflects increased repetition attenuation with learning for the structured sequence, but this was not the case for the pseudorandom sequence (in red). The green clusters show brain regions exhibiting a linear change in BOLD signal for the structured blocks only across the training runs (Z > 2.3, p < 0.05, whole-brain corrected). Both yellow and green designated clusters include the intracalcarine cortex of primary visual cortex. Group-based fMRI analyses report anatomical regions based on Harvard-Oxford Probabilistic Atlas, as part of FSL [13]. (B) BOLD activity map associated with learning a visible second-order conditional visuospatial sequence (Z > 2.3, p < 0.05, whole-brain corrected), obtained when using an equivalent linear contrast to that described above on the dichoptic learning protocol (i.e., comparing the linear trends for structured and pseudorandom sequences in the control fMRI study). Response changes were observed in the angular gyrus (MNI 58 −56 38, Z = 3.91), precuneus (−4 −78 40, Z = 3.89), middle frontal gyrus (24 12 44, Z = 3.93), and superior frontal gyrus (12 28 58, Z = 3.86). Activity was also found in subcortical foci, including mediodorsal and ventral anterior thalamus (bilateral, 2 −14 12, Z = 4.2), extending into the left putamen (−18 8 −2, Z = 3.04). We found no areas exhibiting a linear change in BOLD signal for the structured blocks across the training runs.

In line with prior studies of sequence learning, whole-brain analyses tested for linear effects of exposure to the non-conscious structured second-order conditional (SOC)-based sequence by comparing it against a pseudorandom baseline sequence. Estimates were derived separately for structured and pseudorandom blocks, and these were compared at a higher level of analysis (see Figure 2A legend and Supplemental Information). Activity was observed in a set of regions that included right putamen and pallidum (Montreal Neurological Institute [MNI] 20 4 0, Z = 3.62), right insula (40 −2 −14, Z = 3.59), inferior temporal gyrus (46 −54 −8, Z = 3.78), hippocampus (30 −20 −12, Z = 2.59), lateral occipital cortex (34 −82 −2, Z = 3.39), fusiform gyrus (30 −66 −6, Z = 3.61), lingual gyrus (24 −60 −8, Z = 3.56), and occipital pole (8 −96 0, Z = 3.33), which notably included the intracalcarine cortex of primary visual cortex (6 −88 −2, Z = 3.09). Figure 2A depicts the brain activity maps (in yellow). Learning was also examined by testing linear effects of exposure to the structured sequence alone (Figure 2A, indicated in green), which again showed activity in the intracalcarine sulcus (peak MNI −18 −64 4, Z = 3.3), extending into the lingual gyrus and precuneus. Activity in V1 is thus unlikely to reflect relative differences in novelty of the pseudorandom versus structured sequences within a run, and, as we discuss later, the change in V1 activity predicted behavior on the non-conscious recognition test, which could not be solved on the basis of relative novelty.

Unlike the non-conscious recognition test, where old and new retrieval cues were perceived in the same serial order, there was a visible difference in perceived L-R serial order between structured and pseudorandom blocks during the learning phase (see Supplemental Experimental Procedures), and not all low-level structural properties were equated across the two sequence types. In line with previous studies [15], the serial order of the visual targets (L-R) on unstructured baseline blocks was pseudorandom to facilitate learning of SOC structured sequence. This may have modulated how attention was allocated during learning and hence could account for the observed learning activity in V1. Therefore, a separate control fMRI study was conducted to examine the learning of a SOC sequence under conditions where the four locations were available to visual awareness. All other aspects of the control fMRI study were equated with the dichoptic learning protocol, including the LDT counting task, and learning without motor responses (see Supplemental Experimental Procedures section “fMRI control experiment: Experimental procedures”). Learning the visible sequence was associated with a right fronto-parietal network, which also included subcortical foci in thalamus and left putamen (Figure 2B). These regions were used as a mask to test for learning-related activity in the dichoptic learning protocol. Only the left putamen (−24 10 −2, Z = 3.76, corrected) was associated with learning-related activity under dichoptic masking. Conversely, when the regions that showed learning-related activity during dichoptic masking of the sequence (i.e., primary visual, occipito-temporal cortex, hippocampus, and basal ganglia) were used as a mask to test for learning-related activity associated with the visible sequence, activity was confined to foci in the right putamen (26 4 −10, Z > 2.3, corrected). Again, there was no evidence of additional overlap. Finally, an unpaired t test was performed to compare the parameter estimates for the learning effects associated with conscious visible sequences with those in the dichoptic masking experiment. The results showed that learning-related activity in superior frontal and prefrontal areas was greater with visible than with the masked sequences.

Together, these results indicate that learning-related activity in the dichoptic masking protocol is unlikely to have been driven by perceptual differences between structured and pseudorandom sequences, sensory adaptation, or other non-specific learning effects, because these factors were equated in both fMRI studies, yet the learning-related brain networks did not map across the learning of conscious visible and non-conscious sequences. We present additional information from the non-conscious recognition test below in support of this interpretation.

### Behavioral Evidence for Non-conscious Recognition Memory

Non-conscious recognition memory was first examined using a contrast based on confidence ratings associated with all old and all new stimuli (retrieval cues). Although the proportion of correct “old”/“new” discriminations was at chance (mean = 0.51 [SEM = 0.01]; t_(17)_ = 0.68, p > 0.05), the memory ratings associated with all “old” and all “new” retrieval cues were significantly different (mean “old” = 2.64 [SEM = 0.04], mean “new” = 3.16 [SEM = 0.07]; t_(17)_ = −7.95, p < 0.001; Figure S2), indicating recognition of the non-conscious sequence. This result is in keeping with recent behavioral evidence of recognition memory without visual awareness [16, 17] and aligns with other behavioral evidence that has examined the learning of non-conscious first-order sequences [18].

Additional analyses were based on receiver operating characteristics (ROC) of type 1 performance (i.e., sensitivity to “old”/“new” retrieval cues) and type 2 performance (i.e., how memory confidence relates to “old”/“new” discriminations), and also type 1 d′ and type 2 meta-d′ [19] (i.e., the efficacy with which observers’ confidence ratings discriminated between their own correct and incorrect memory decisions). Type 1 sensitivity for “old”/“new” discrimination was at chance (i.e., for sensitivity based on the area under the type 1 ROC: t_(17)_ = 1.45, p = 0.17, mean = 0.52, SEM = 0.01, chance = 0.5; for type 1 d′: t_(17)_ = 0.59, p = 0.57, mean = 0.03, SEM = 0.05, chance = 0), whereas type 2 sensitivity was significantly above chance (for sensitivity based on the area under the type 2 ROC: t_(17)_ = 5.79, p < 0.001, mean = 0.63, SEM = 0.02, chance = 0.5; for type 2 meta-d′: t_(17)_ = 4.90, p < 0.001, mean = 0.9, SEM = 0.18, chance = 0) (see Figure S3, Supplemental Results, and Tables S1 and S2). These results are consistent with recent evidence indicating that metacognitive processes (e.g., related to perceptual decision making or memory) can be successfully deployed even when type 1 sensitivity is null [20, 21], i.e., under conditions that are independent of visual awareness [22]. The current study and other recent studies set precedents that require additional experimental work to examine the relationship between visual awareness and higher-order cognition.

There was no significant difference in the manual response times (RTs) to “old” and “new” retrieval cues (mean RTs, 2018 ms versus 2119 ms, respectively; t_(17)_ = 0.86, p = 0.40). Therefore, any increase in perceptual fluency (i.e., the perceived speed of processing an item), induced by prior exposure, did not lead to faster identification times (response priming), which suggests that response fluency did not serve as a basis on which to recognize prior occurrence (cf. [23]). Notably, prior studies have reported RT differences between old and new retrieval cues based on visible sequences (e.g., [24]). The absence of such priming effects in the current study may be because motor-based responses were not performed during learning.

### Relevance of Learning-Related Activity for Subsequent Non-conscious Recognition Memory

The relevance of learning-related activity for knowledge of the trained SOC sequence was examined using a robust multiple regression analysis based on the magnitude of the corrected blood-oxygen-level-dependent (BOLD) signal change during learning within the calcarine sulcus and six other functional regions-of-interest (ROIs) (hippocampus, inferior temporal sulcus, insula, putamen, fusiform cortex, and lingual gyrus). Each was entered as a variable in a multiple linear regression using Huber’s method of correction for outliers against the magnitude of non-conscious recognition memory (old minus new mean confidence ratings). Learning-related activity within the calcarine sulcus predicted behavior on the non-conscious recognition test (β = 0.35, p < 0.05), whereas the activity estimates from the six other ROIs were not significant predictors of non-conscious recognition memory (all p’s > 0.21). If learning-related activity in V1 had merely reflected low-level sensory adaptation effects, low-level structural or relative novelty differences, and/or perceived differences in serial order between the structured and pseudorandom sequences, then the V1 signal change during learning should not have predicted recognition memory, because structural and perceived differences were equated between old and new retrieval cues used on the non-conscious recognition test (Table S3).

### Neural Substrates of Non-conscious Recognition Memory

**Figure 3:**
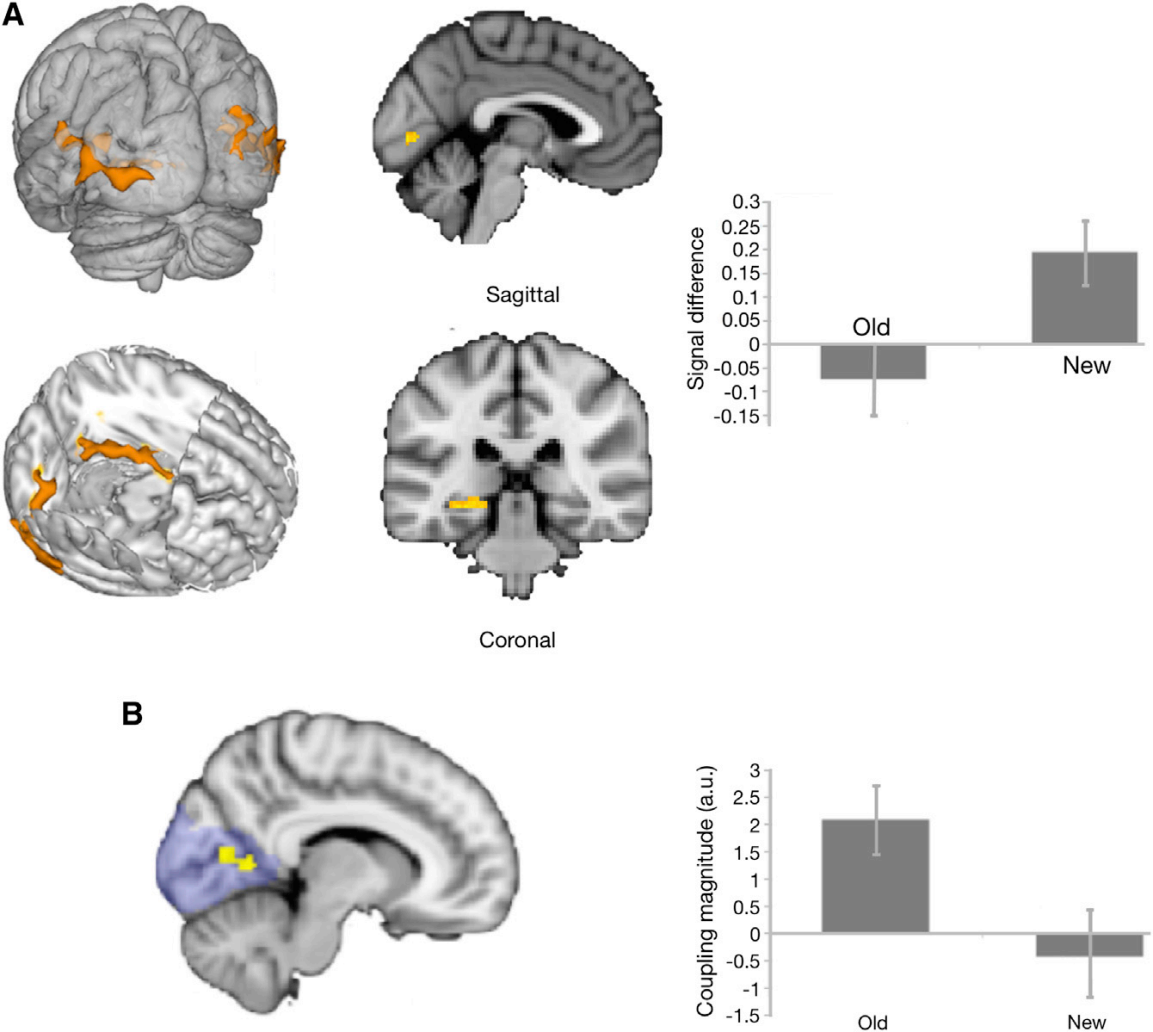
BOLD Responses and Functional Connectivity Associated with Non-conscious Recognition Memory (A) BOLD responses associated with non-conscious recognition memory (for behavioral results, see Figures S2 and S3 and Tables S1 and S2). Left: brain regions showing BOLD activity for the contrast between old < new sequences (Z ≥ 2.3, p < 0.05, whole-brain corrected). Right: plot of the signal difference in these regions associated with recognition of the non-conscious sequence. Old and new retrieval cues differed only in terms of the monocular target sequence, whereas the perceived serial order associated with the old and new retrieval cues was the same. As an example, the perceived serial order of a non-conscious old cue specified across monocular (1, 2, 3, 4) and eye-of-origin ([L] = left; [R] = right) locations, 3[L]-4[R]-1[L]-2[R]-4[R]-3[L], was matched to a non-conscious new cue specified across monocular and eye-of-origin locations, 1[L]-4[R]-3[L]-2[R]-4[R]-1[L] (see also Table S3). Error bars correspond to SEM. (B) Results from a psychophysiological interaction-based analysis that examined functional connectivity associated with a hippocampal-based seed voxel drawn from the responsive voxels in the non-conscious old < new recognition memory-based contrast. Functional coupling between the hippocampus and the intra-calcarine cortex (V1, indicated in yellow) was modulated as a function of whether the retrieval cue on the recognition test was old or new (Z > 2.3, p < 0.05, corrected for the occipital mask in blue shading). The graph on the right shows that the magnitude of the functional coupling was higher for old relative to new retrieval cues. Error bars correspond to SEM.

First, we assessed BOLD activity for the old < new contrast using a whole-brain mass-univariate analysis. Non-conscious recognition-related activity was observed in a visual cluster with a peak in the lingual gyrus (MNI −20 −50 −4, Z = 3.7), which extended into the intra-calcarine sulcus in V1 (−4 −80 2, Z = 2.79). Left and right lateral occipital cortex (LOC) also exhibited activity, with peaks in left inferior LOC (−52 −76 −6, Z = 3.64) and right superior LOC (28 −88 26, Z = 4.39). Notably, the old < new effect was also found in the left hippocampus (−22 −38 0, Z = 3.49). Figure 3A depicts the brain activity maps. No significant brain changes were found in the old > new contrast.

Second, we performed the same analyses using functional ROIs derived from the intracalcarine cluster observed during the learning phase, with voxelwise correction for multiple comparisons (p < 0.05). Notably, learning and non-conscious recognition-related activity overlapped in the intracalcarine cortex (MNI −0 −80 2, Z = 3.17 and −14 −68 8, Z = 3.29). This overlap suggests that V1 activity can occur even when there are no low-level structural differences between the sequences that form the basis of the contrast, as compared to the structured versus pseudorandom block-based linear contrast in the learning phase.

Third, following previous studies that identified the conscious recall of visible grating pairs with hippocampal-V1 coupling [25] and coupling between the hippocampus and early visual cortex with implicit statistical learning of visible stimuli [26], we tested for functional connectivity between hippocampus and V1 during non-conscious recognition by means of psychophysiological interaction (PPI) analysis. A mask of the hippocampus was drawn from the responsive voxels in the old < new contrast described above and used to define the seed region’s time course. Given our a priori interest in V1, this analysis used a region-of-interest approach with an occipital mask. Figure 3B illustrates the PPI results. A significant cluster was found in left V1 (MNI 10 −70 12) that, critically, coincided with the intra-calcarine cortex (Z > 2.3, p < 0.05 corrected for the occipital mask). This V1 cluster exhibited increased functional coupling with the hippocampus during the presentation of old relative to new retrieval cues. The same result was observed in the adjacent intra-calcarine area of the right hemisphere (Z > 2, p < 0.05, corrected). Individual measures of functional connectivity did not correlate with non-conscious recognition memory behavior (p > 0.05).

Finally, the link between human V1 and calcarine sulcus is well described [27], and activity in the intracalcarine cortex and its vicinity coincided with probabilistic V1. Nonetheless, given our a priori interest in V1, a functional localizer was administered in a separate fMRI scan to map regions in early visual cortex that were associated with visual stimulation at the monocular locations. V1 was automatically defined on each individual structural scan by applying the method developed by Hinds and colleagues [28]. This method is implemented in Freesurfer [29], in order to predict the location of the stria of Gennari—an anatomical marker of primary visual cortex—with reference to cortical surface topology, which is demonstrated to be as accurate as a retinotopic mapping [30]. Individual masks with a probability of 0.99 of occurring in V1 were obtained on each observer to mitigate uncertainties about architectonic borders. Individual masks were then derived for the intersection of active voxels in visual cortex seen on the localizer and the V1 area derived from Freesurfer. These masks were used to extract individual parameter estimates for the old < new contrast. A one-sample t test showed that the old < new recognition effect was significantly different from chance (t_*(*17)_ = 2.14, p < 0.05).

### Behavioral Significance of Activity Associated with Non-conscious Recognition Memory

This issue was examined by using the same robust multiple-regression-based method described earlier, but with recognition fMRI-based ROIs in the calcarine sulcus, hippocampus, and right occipital cortex. The difference in activity within the calcarine sulcus predicted behavior in the non-conscious recognition test (β = 0.31, p < 0.05). Likewise, activity in the right occipital cortex also predicted behavior in the non-conscious recognition test, with a negative association (β = −0.47, p < 0.05), whereas the activity estimate from the hippocampus was not a significant predictor (p = 0.32). These results suggest that activity in V1 and the occipital cortex both predicted non-conscious recognition memory but that only the magnitude of the old < new response in V1 was linked with improved non-conscious recognition memory. One interpretation of these associations is that non-conscious recognition processes may operate not only by local signal differences in V1 activity but also by concurrent inhibition in connected occipital structures that respond to the monocular stimuli. Push-pull processes of this type may optimize discrimination processes in the service of non-conscious recognition memory, but definitive hypotheses about possible gating mechanisms involving regional activity within occipital cortex associated with non-conscious recognition memory await further investigation.

## Discussion

Learning-related activity occurred in cortical and subcortical regions, including primary visual cortex, as part of a temporo-occipital and basal ganglia network. In contrast to the much-studied serial reaction time task used to investigate sequence learning [31], observers did not direct motor responses or motor attention to the visual stimuli. Therefore, activity reflected complex learning in the absence of mechanisms related to motor responding, and the overlap with several regions identified in visuomotor sequence learning [4, 15] is thereby consistent with common mechanisms of perceptual and motor learning [32]. By contrast, V1 activity has not been seen in prior investigations of explicit (conscious) and implicit (non-conscious) human visuomotor sequence learning of visible stimuli, nor was it observed in our control fMRI learning study based on a visible SOC sequence. V1 has however been implicated with coding simple serial associations and timing information in an experimental animal mouse model, when studied using stimuli (visible gratings) designed to induce activity in V1 [7].

Unlike later visual cortical areas such as the perirhinal and infero-temporal cortex, there is little experimental data to implicate V1 in recognition memory. Most notably, recent work in a mouse model has linked V1 with a form of recognition memory based on discriminations between visible old and novel oriented gratings [6, 33]. We extend these observations by showing that early visual cortices in humans, including V1, a region implicated in perception and perceptual learning of low-level features [34], is associated with learning and subsequent non-conscious recognition memory of a repeating complex visuospatial sequence. Importantly, by broadening the scope of investigation to high-level memory-guided behavior, we have demonstrated that experience-dependent changes in V1 can (1) occur even when the spatial location of targets is masked from visual awareness, (2) arise over an hour rather than several days (cf. [1]), (3) operate in the absence of reward or punishment [35], and (4) generate a signal that predicts behavior (i.e., recognition confidence) outside of the original conditions of (perceptual) learning (cf. [36]).

Isolating non-conscious processes in recognition memory has proved difficult to achieve. One potential reason is that previous behavioral paradigms have administered visible stimuli at encoding and as retrieval cues ([8, 37]; but see [16]). The key advantage of our protocol is that activity in visual cortex and the hippocampus were not mediated by modulation due to conscious perceptual expectations (related to the four monocular locations) [38], the reinstatement of episodic information [39], or other old/new differences in processes—such as evaluation and decision making—that operate “downstream” of conscious retrieval. Furthermore, the non-conscious recognition effect seen here is distinguished from other non-conscious forms of memory, such as repetition priming, because repetition-related occipital fluency effects (1) are often unrelated to behavior [40], (2) if present, only prime a particular response such as identification [41], rather than supporting behavior outside of the original study context (i.e., discrimination based on old/new recognition confidence ratings), and (3) involve behavioral phenomena that are considered to be short lived [42], rather than operating 15–20 min after initial learning, as in the current study.

The enhanced functional coupling observed between V1 and the hippocampus during non-conscious recognition is noteworthy (1) given evidence from experimental animal models that implicate the entire ventral visual-to-hippocampal stream in memory for visible items [43] and (2) in light of data in humans wherein hippocampal activity has been aligned with (conscious) memory strength rather than recollection and familiarity [44] and has been shown to be sensitive to the distinction between (visible) old versus novel stimuli [45]. Unlike in previous studies, the hippocampal activity seen here was not attributable to a general novelty signal, a difference in familiarity, or conscious memory strength (cf. [46, 47]), because the new retrieval cues were not based on novel stimuli with respect to the learning phase (cf. [16]), and the serial order of the (binocular) perceived two-location old and new retrieval cues was equally familiar. Old and new retrieval cues were distinguished only in terms of their unique non-conscious serial order, specified at the masked four monocular locations. More broadly, the observed hippocampal activity is notionally consistent with the proposal that relational complexity, rather than mechanisms associated with conscious perception, modulates hippocampal engagement [48].

In summary, our results identify experience-related plasticity in a visual area as early as human V1 with behavior on a recognition memory test that excluded mnemonic mechanisms related to visual awareness. The results are central to broadening the scope of theoretical work on the role of early visual cortex in learning and memory, and go beyond dominant dual-process (episodic) models of recognition memory that have centered on explaining the conscious retrieval of episodic traces or familiarity mediated by the medial temporal lobe.

## Author Contributions

Conceptualization, C.R.R. and D.S.; Methodology, C.R.R.; Software, C.R.R. and D.S.; Formal Analysis, C.R.R. and D.S.; Investigation, C.R.R., S.K.A., and C.A.A.; Resources, C.R.R.; Writing – Original Draft, C.R.R.; Writing – Review & Editing, C.R.R., D.S., and C.K.; Visualization, C.R.R. and D.S.; Supervision, C.R.R. and D.S.; Project Administration, C.R.R.; Funding Acquisition, C.R.R., C.K., and D.S.

## Supplemental Information

Document S1. Figures S1–S3, Tables S1–S3, Supplemental Experimental Procedures, and Supplemental Results

Movie S1. Dichoptic Presentation Protocol Used During Learning and Test Phases, the Location Awareness Test, and the Supplemental Eye Movement Control Experiment, Related to Figure 1 The movie shows how monocular locations were masked from visual awareness by the use of dichoptic viewing through a prism-based stereoscope inside the bore of the MRI scanner. Head position and eye position were fixed throughout all fMRI data acquisition stages. The foreground two-location placeholder corresponds to the image perceived when the four locations were viewed through the prism-based stereoscope. First, a 12-element second-order sequence was repeatedly presented across four monocular locations on a computer screen. Note the perceived (two-position) binocular sequence contains no eye-of-origin information from the four monocular stimulus locations in the non-conscious sequence (separately verified using data analyzed using signal detection theory). Stimuli presented in locations 1 and 3 were perceived on the left, whereas stimuli presented in locations 2 and 4 were perceived on the right. Second, an example six-item “old” recognition retrieval cue is shown (i.e., in the format of presentation used on the dichoptic recognition test) and followed by an illustrative parallel presentation of six-item “old” and “new” recognition retrieval cues to depict how the perceived serial order was matched between these two types of retrieval cue. As the illustration of the matching of old and new retrieval cues depicts, the perceived serial order associated with each old and new retrieval cue were equated so that recognition memory could be based only on sensitivity to serial order of the old/new non-conscious sequence (specified across the four monocular positions). In all example trial sequences, the rate of stimulus presentation is slowed for illustrative purposes (see Supplemental Experimental Procedures for the SOA values used in the experiments).

Document S2. Article plus Supplemental Information
