# Supplementary material for: Learning and Recognition of a Non-conscious Sequence of Events in Human Primary Visual Cortex
Source: Curr Biol. 2016 Mar 21;26(6):834–41. doi: 10.1016/j.cub.2016.01.040 (PMC4819512; doi:10.1016/j.cub.2016.01.040)
Supplement: Document S1. Figures S1–S3, Tables S1–S3, Supplemental Experimental Procedures, and Supplemental Results [file mmc1.pdf]

Current Biology, Volume *26*

Supplemental Information

Learning and Recognition of a Non-conscious Sequence of Events in Human Primary Visual Cortex

Clive R. Rosenthal, Samantha K. Andrews, Chrystalina A. Antoniades, Christopher Kennard, and David Soto

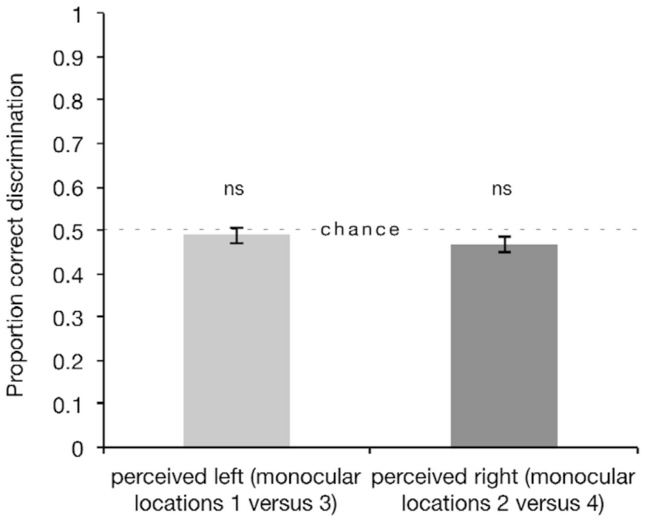

Figure S1 is related to Figure 1 (test phase, section 2c: location awareness test). Perceptual sensitivity on the location awareness test (LAT) for the four monocular locations associated with the non-conscious sequence. Participants were unable to identify the correct monocular locations of the perceived visual targets, despite extensive the perceptual training associated with repeated exposure to the non-conscious sequence during learning and recognition phases, and later instruction on the mapping between the four-monocular locations and two perceived locations, before responding on the LAT. Null sensitivity for monocular location information held when d' – a signal detection theory based measure of perceptual sensitivity – was analyzed at both perceived locations. Error bars correspond to standard error of the mean.

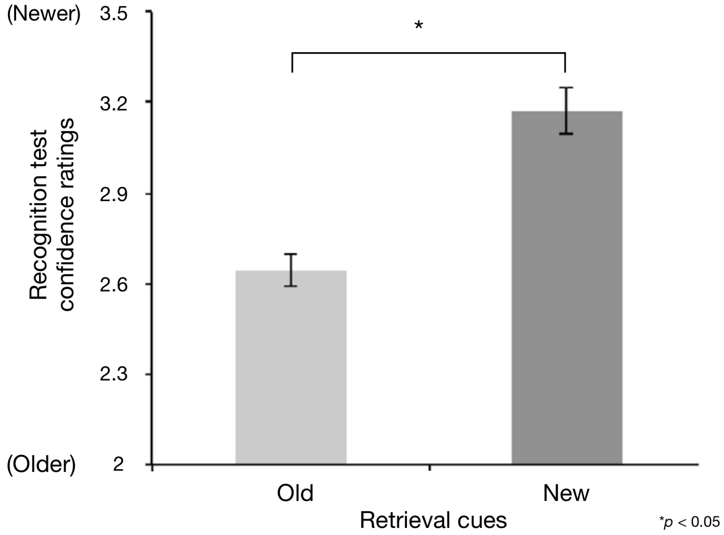

Figure S2 is related to Figure 1 (test phase, section 2b: non-conscious recognition test) and Figure 3 (behavioural results predicted by BOLD activity in V1). Recognition memory for the non-conscious sequence. Mean confidence ratings associated with the non-conscious 'old' second-order conditional and non-conscious 'new' second-order conditional

based retrieval cues were significantly different ($t_{(17)}$ = -7.95, $p < 0.001$, repeated measures t-test). Error bars correspond to standard error of the mean.

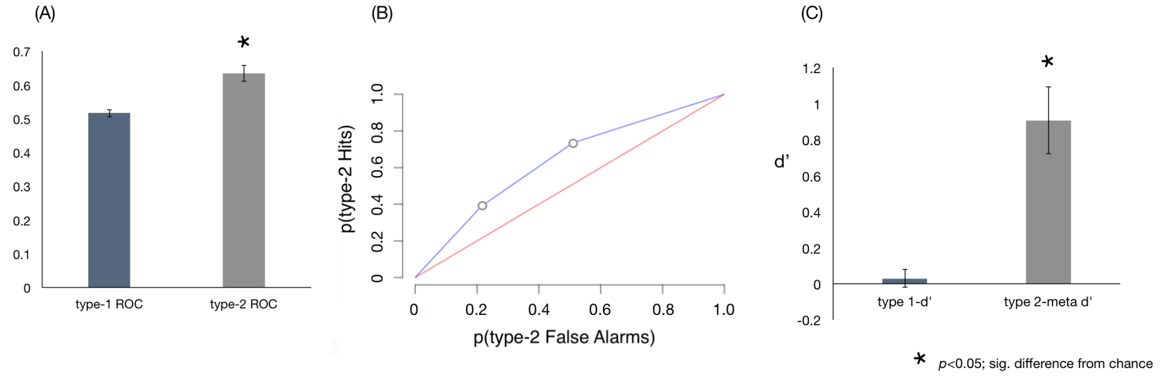

Figure S3 is related to Figure 1 (test phase, section 2b: non-conscious recognition test) and Figure 3. Type-1 and type-2 sensitivity analyses of recognition memory performance (A). Area under the ROC curve (AuC) for type-1 and type-2 performance. Type-1 sensitivity for 'old'/'new' discrimination was at chance (i.e., for sensitivity based on the area under the type-1 ROC: $t_{(17)}$=1.45, p=0.17, mean=0.52, s.e.m.=0.01, chance=0.5), whereas type-2 sensitivity was significantly above chance (for sensitivity based on the area under the type-2 ROC: $t_{(17)}$=5.79, p<0.001, mean=0.634, s.e.m.=0.02, chance=0.5) **(B)** type-2 receiver-operating-characteristic (ROC) curve (C) Analyses of d' for type-1 and type-2 performance (meta-d'). Type-1 d' was at chance ($t_{(17)}$=0.59, p=0.57, mean=0.03, s.e.m=0.05, chance=0), whereas type-2 meta-d' was significant ($t_{(17)}$=4.90, p<0.001, mean=0.9, s.e.m.=0.18, chance=0). Error bars correspond to standard error of the mean.

Supplemental Tables

Table S1 is related to Figure 1 (test phase, section 2b(ii): behavioural results from non-conscious recognition test old/new responses) and Figure 3. Proportion of 'old'/'new' responses across old (trained) and new (untrained) retrieval cues (stimuli)

	Old (trained) retrieval cue	New (untrained) retrieval cue
'Old' response	0.30	0.29
'New' response	0.20	0.21

Table S2 is related to Figure 1 (test phase, section 2b(iii): behavioural results from non-conscious recognition test confidence ratings) and Figure 3. Proportion of confidence responses across old and new retrieval cues

	Least certain	Fairly certain	Certain
Old retrieval cue	0.21	0.14	0.15
New retrieval cue	0.18	0.18	0.14

Table S3 is related to Figure 1 (test phase, section 2b(i): stimulus materials used on non-conscious recognition memory test) and Figure 3. **Masked locations of old and new retrieval cues presented on the non-conscious recognition memory test.** Old and new retrieval cues were based on two different 12-element second-order conditional sequences (SOC1 and SOC2). The old or new status of each six-item sequence derived from the respective 12-element sequences was determined by training on the dichoptic sequence learning protocol (SOC1 or SOC2). Critically, perceived binocular locations for SOC1 and SOC2 are identical across matched pairs of the six-item recognition retrieval cues, and all perceived and non-conscious structural properties were equated, along with low-level stimulus properties such as the laterality of stimuli at eye-of-origin. Masked locations 1, 2, 3, 4, read from left to right for masked four-location placeholder array. L = Left placeholder; R = Right placeholder.

Companion old and new retrieval cues used on the non-conscious recognition memory test		
Monocular locations of non-conscious cues (SOC1)	Monocular locations of non-conscious cues (SOC2)	Locations of perceived retrieval cues (i.e., perceived old and new retrieval were equated for serial order and location)
3 4 2 3 1 2	1 4 2 1 3 2	L R R L L R
4 2 3 1 2 1	4 2 1 3 2 3	R R L L R L
2 3 1 2 1 4	2 1 3 2 3 4	R L L R L R
3 1 2 1 4 3	1 3 2 3 4 1	L L R L R L
1 2 1 4 3 2	3 2 3 4 1 2	L R L R L R
2 1 4 3 2 4	2 3 4 1 2 4	R L R L R R
1 4 3 2 4 1	3 4 1 2 4 3	L R L R R L
4 3 2 4 1 3	4 1 2 4 3 1	R L R R L L
3 2 4 1 3 4	1 4 2 1 3 2	L R R L L R
2 4 1 3 4 2	2 4 3 1 4 2	R R L L R R
4 1 3 4 2 3	4 3 1 4 2 1	R L L R R L
1 3 4 2 3 1	3 1 4 2 1 3	L L R R L L

Supplemental Experimental Procedures

Participants

Eighteen undergraduate students at the University of Oxford were recruited (mean age of 21.9 years; nine female). All participants gave informed written consent to take part in accordance with the terms of approval granted by the local research ethics committee, the principles expressed in the Declaration of Helsinki, and were naive to the purpose of the

experiment. All participants reported normal or corrected-to-normal vision, no history of neurological disease, and no other contraindications for MRI.

Dichoptic presentation and visual stimuli

Presentation of the visual targets involved the use of dichoptic presentation using prism-based eyeglasses, detailed by Schurger [S1] and illustrated in Figure 1 and in Supplemental Movie 1. Participants lay supine on the scanner bed, and foam pads were used around the head to minimize head movements. Six pairs of MRI-compatible spectacles, fitted with prism lenses from 2–12 diopters in steps of 2, were available to cover disparities of 1.5, 2.3, 3.4, 4.5, 5.7, and 6.8 degree of visual angle and individual differences in fusion range. This method of dichoptic presentation was used because it eliminated crosstalk between each eye-of-origin, equated the luminance arriving at each eye, and enabled binocular fusion to be sustained for long periods [S1, S2].

The stability of binocular fusion inside the scanner was tested before each block of trials during the learning phase, the recognition test phase, the functional localiser, and on the location awareness test – the accuracy of responding served as an additional test of the efficacy of masking [S3, S4]. The test of binocular fusion involved the presentation of a short sequence of visual targets at the four fixed monocular locations (two at each eye-of-origin). Participants were required to provide an accurate online verbal report from inside the scanner of the perceived location of each of target presented at each monocular location. All participants reported only seeing two targets and reported the locations of the perceived targets accurately throughout all stages of the experiment.

All visual stimuli were presented on a computer screen (resolution: 1024 x 768 pixels), and were viewed via a mirror attached to the head coil of the MRI scanner. Each target was presented for 2000 ms at the centre of one of four monocular locations circumscribed by two horizontally aligned and isoluminant figures-of-eight (read from left to right, placeholders 1 and 2 were in the left figure-of-eight and placeholders 3 and 4 were in the right figure-of-eight). When viewed through the prism-based stereoscope, the placeholders were perceived as a single, fused, and centrally positioned figure-of-eight, aligned with the horizontal meridian. Visual targets presented at monocular locations 1 and 3 appeared within the left placeholder of the fused figure-of-eight (binocular left targets), whereas targets presented at monocular locations 2 and 4 appeared within the right placeholder (binocular right targets). Hence, on any single trial, the target stimulus was presented to the separate and independent field of view of one eye, within one of the monocular locations masked by dichoptic fusion. The eye-of-origin of visual input for each target was thereby continuously masked from visual awareness; i.e., location information for the target stimuli and thus the target visuospatial sequence were masked from visual awareness. The efficacy of visual masking was independently assessed on a location awareness test (LAT) (see below). This method of masking the monocular sequence of targets from visual awareness was continuous and did not rely on subjective report.

Behavioural tasks: Design, Materials and Procedures

The experiment was conducted in two separate main phases (Fig. 1): (1) Learning phase (dichoptic sequence learning protocol): participants were presented with a single repeating 12-element non-conscious (monocular) visuospatial sequence, based on a second-order conditional rule, and displayed at four monocular locations on a computer monitor; (2)

Test phase (a) a sequence awareness questionnaire: participants responded immediately after the learning phase to indicate whether they had detected any regularities in the sequence of stimuli; (b) dichoptic non-conscious recognition memory test: here, observers (i) viewed either an old/studied or a new/non-studied retrieval cue comprised of a sequence of six visual targets, which were presented in the same way as on the dichoptic sequence learning protocol; (ii) responded to indicate whether each retrieval cue was either old or new, with respect to the learning phase; and, (iii) rated their confidence associated with each old/new response on a six-point scale (see below). Importantly, the old and new retrieval cues differed only in terms of the masked serial order of the non-conscious sequence, and followed an identical perceived serial order (Fig. 1, Test phase; Table S2); and, (c) location awareness test (LAT): the LAT was administered to re-assess the efficacy with which location information for the target four-location visuospatial sequence was masked from visual awareness, following repeated (perceptual) exposure to the monocular sequence of stimuli during the learning and test phases (Fig. 1c).

Participants completed a practice version of the dichoptic sequence learning protocol and all four phases inside the scanner (total duration: ~2.5 hr). Participants were instructed to avoid making eye movements during learning and tests phases and during the LAT. fMRI data were acquired during phases 1 and 2b. fMRI data were also acquired during functional localiser scan for the monocular locations (see below). All of the behavioural tasks were implemented and administered using E-prime Pro (version, 2.0; Psychology Software Tools, Inc. Pittsburgh, PA).

Learning phase: dichoptic sequence learning protocol and large diameter target counting task

Participants were not informed about the repeating sequence during the learning phase, and were naive to the non-conscious monocular location of each target – separately, we have demonstrated that an intentional orientation to the sequence does not modulate learning or facilitate the detection of the non-conscious sequence [S3]. Visual targets consisted of white circles of two sizes – a standard (5 mm diameter) and a large diameter target (LDT; 8 mm diameter) – presented within one of the four monocular locations (Fig. 1) for 2000 ms.

Targets were perceived at one of two locations, and participants were asked to discriminate between the standard (5 mm diameter) and large diameter targets (LDTs; 8 mm diameter), in order to maintain a cumulative count of the number of LDTs on each block and perform within 5% accuracy [S3, S5]. Presentation of LDTs occurred on 17-35% of trials on both sequence and pseudorandom baseline blocks (see below) – the order of the LDTs was random within a block and set at a proportion to ensure ceiling level performance. The LDT counting task was performed concurrently with the dichoptic sequence learning protocol. On-screen feedback - in the form of percentage under- or over-estimation - at the end of each block of trials was provided immediately after entering a value on the LDT counting task.

Notably, performance on the LDT counting task during the learning phase provided an additional and independent source of data regarding the stability of binocular fusion inside the scanner, because ceiling performance was dependent on stable fusion [S3, S4]. An initial practice block comprised of 30 trials was administered inside the scanner to ensure task comprehension and accurate performance on the LDT counting task. During the learning,

blocks were separated by a 30 s response period to allow a value on the LDT counting task to be entered by the participant.

Each sequence block was comprised of eight repetitions of the same 12-element second-order conditional sequence (SOC) specified at the four monocular locations. Two SOCs were counterbalanced across participants to control for sequence-specific effects (SOC1: 3-4-2-3-1-2-1-4-3-2-4-1; SOC2: 3-4-1-2-4 3-1-4-2-1-3-2; positions 1-4 are read from left to right of the masked four-location array, with the spatial locations corresponding to numeric values). Half of the observers studied SOC1 and the other half studied SOC2. Each SOC sequence was equated along dimensions that ensured learning was directed towards the SOC rule; namely, simple frequency of location, laterality, frequency of first-order transition, reversal frequency (e.g., 1-2-1), and rate of full coverage. The two SOC sequences differed only at the level of serial order of three or more consecutive positions within the sequence, where, at the lowest structural level, the ability to predict a target position is dependent on learning two preceding monocular target positions. Unlike a first-order sequence, performance cannot improve from learning the frequencies of individual positions or pairs of positions. Binocular perception of the visual targets during the learning phase was consistent with the following sequences of left(L)/right(R) perceived locations: SOC1: L-R-R-L-L-R-L-R-L-R-R-L; and, SOC2: L-R-L-R-R-L-L-R-R-L-L-R.

The learning phase was implemented in three runs of fMRI acquisition, separated by breaks of approximately 5 min (Fig. 1). Runs 1 and 2 were comprised of four sequence blocks (S; 100 trials/block) and two baseline blocks (R; 50 trials/block) (SSRSRS). Run 3 was comprised of five sequence blocks and two baseline blocks (SSRSRSS). An additional sequence block was included at the end of the run to facilitate learning of the target non-conscious sequence.

The structure of the blocks during the learning phase was designed to facilitate rapid acquisition of sequence-specific SOC knowledge. The serial order of the visual targets on the baseline (unstructured) blocks was pseudorandom, and was constrained so that the same monocular location did not appear consecutively and targets appeared with equal frequency at each of the four monocular locations. Therefore, the serial order of structured and pseudorandom blocks were visibly different (i.e., at the perceived two-location L-R locations). We elected to use a pseudorandom sequence, instead of a non-trained second-order conditional sequence on baseline blocks to facilitate learning of the target SOC sequence blocks.

Sequence awareness questionnaire

Awareness associated with learning phase was tested on a post-learning phase awareness questionnaire administered during the break between learning and test scanning phases (see below). Each participant was instructed to select one of the following five propositions: 1 = "The sequence of stimuli was random"; 2 = "Some positions occurred more often than others; 3 = "The movement of the stimuli was often predictable"; 4 = "The same sequence of stimuli would often appear"; and 5 = "The same sequence of stimuli occurred throughout the experiment" [S6].

Dichoptic non-conscious recognition memory test

The dichoptic recognition memory test was administered approximately 15-20 minutes after completion of the dichoptic sequence learning protocol. Each trial on the recognition test was comprised of three stages. **(i)** Participants were shown either an old or a new retrieval cue (six-element sequences; 12 old and 12 new) in a random order (7.2 s; Fig. 1b). Stimuli that comprised the retrieval cue were presented and viewed in the same way as on the dichoptic sequence learning protocol. **(ii)** One second after the offset of each retrieval cue, a prompt appeared for the participants to identify the sequence as either 'old' or 'new' (8 s) (with respect to the learning phase). It was emphasized to participants that a proportion of the sequences had been studied, and an 'old' response should be given if they felt any sense that the retrieval cues were at all familiar, using whatever minimal information was available to aid accurate identification of the old sequences. **(iii)** Participants then provided a value on a confidence rating scale to indicate the confidence in their response (8 s) [S7]: for retrieval cues designated as, old: 1 = "I'm certain that this fragment was part of the training sequence"; 2 = "I'm fairly certain that this fragment was part of the training sequence"; 3 = "I believe that this fragment was part of the training sequence"; for retrieval cues designated as new: 4 = "I believe that this fragment was not part of the training sequence"; 5 = "I'm fairly certain that this fragment was not part of the training sequence"; 6 = "I'm certain that this fragment was not part of the training sequence." The scale was designed to maximise response distance between old and new retrieval cues, and encourage participants to consider the scale in full.

Twelve six-element retrieval cues (starting from each ordinal position of the 12-element SOC sequence for six consecutive locations) were generated from SOC1 and 12 six-element recognition sequences were generated from SOC2 (see Table S3). These were assigned as old and new retrieval cues in accordance with the counterbalancing described earlier. For each old retrieval cue, the first predictable target occurred after 2.4 s (i.e., the onset of the third target in each six-element sequence); correspondingly, the third stimulus provided the first potential source of discrimination between the old and new retrieval cues (i.e., on the basis that the non-conscious second-order conditional subsequences were all unique to the trained SOC).

The non-conscious recognition memory test was implemented as a slow event-related design (24.2 s for each trial), with variable inter-item lags between each trial to permit efficient segregation of the hemodynamic responses. Manual key presses on one of four keys mapped to the right hand were used to indicate the number of LDT targets presented on the immediately preceding block of trials, during the 30 s count period between blocks of trials. The mapping was implemented to minimise and equate key presses, as far as possible, across each response period. Manual responses were recorded using a fibre optic Lumina Response Pad (LU444-RH, Cedrus Corporation, San Pedro, USA) connected to a PC running E-Prime Pro.

There are two remarkable features of this recognition test. First, the monocular spatial information necessary to distinguish old retrieval cues (e.g., 3-4-1-2-4-3) from new retrieval cues (e.g., 1-4-3-2-4-1) was not available to visual awareness (as confirmed by signal detection theory based analyses of performance conducted on the results from the location awareness test, which was administered after the recognition test). Second, the old and new retrieval cues were both perceived in the same binocular serial order. In particular, for the two example sequences, old and new retrieval cues were perceived as the same, L-R-L-R-R-L, binocular sequence, and so differed only in terms of the masked serial order of the respective old and new second-order conditional sequences (Table S3).

An additional important consequence of matching the structural properties of the old and new retrieval cues – i.e., simple frequency of location, laterality, first-order transition frequency, reversal frequency, presence of second-order conditional serial dependencies and rate of full coverage of the four locations – is that it provided an effective basis on which to study neural and behavioural effects related to recognition under conditions that did not reflect 'old'/'new' differences in the spatial allocation of attention or low-level old-new differences, such as laterality at the eye-of-origin. As an example of the latter, for old and new retrieval cues perceived as, L-R-L-R-R-L, the underlying non-conscious old sequence – 3-4-1-2-4-3 – and new sequence – 1-4-3-2-4-1 – were equated in terms of laterality from each eye-of-origin (3 positions from the left eye-of-origin; 3 positions from the right eye-of-origin).

To summarise, this setup allowed us to base the non-conscious recognition test on a contrast between the studied non-conscious sequence and an non-studied/untrained non-conscious (second-order conditional) sequence that was equated in terms of the perceived serial order and all other structural dimensions of the sequence, apart from the four-location monocular serial order of the non-conscious second-order conditional retrieval cues. The number and six-element format of retrieval cues corresponds with established studies of sequence learning based on the use of a visible 12-element SOC [e.g., S8]. Hence, the recognition memory test assessed observers' sensitivity to the non-conscious second-order conditional properties acquired during the dichoptic sequence learning phase.

Location Awareness Test (LAT)

Visual targets were viewed through the prism-based stereoscopic setup inside the scanner, in the same way as during the dichoptic sequence learning and recognition test phases. The mapping between monocular targets and their appearance at the two perceived locations was explained to the participants in order to encourage accurate trial-by-trial forced-choice responses, and improve sensitivity to even partial knowledge of the location of the monocular stimuli, because this could serve as a basis for accurate detection of the location of each monocular stimulus. Forced-choice responses were performed for targets that appeared within the left binocular location (i.e., choose between masked locations 1 and 3) and for targets that appeared at the right binocular location (i.e., choose between masked locations 2 and 4). Targets remained on screen until a manual response was entered on the response pad, and were separated by a 200 ms ISI. Two blocks of forced-choice probe trials were used to test perceptual sensitivity at all four monocular locations on each block; all four monocular locations were represented equally on the LAT.

An inability to identify masked locations provided evidence that the locations of stimuli presented at the four monocular locations – and thus information necessary to consciously learn the SOC associations specific across eye-of-origin – were masked from visual awareness. More generally, for a 12-element SOC, such location information would need to be continuously available at each of the four monocular locations in order for the sequence to be learned in the same way as on a standard serial reaction time task, which is typically used to investigate conventional somatomotor "implicit" sequence learning at four visible locations [e.g., S9, S10, S11].

Functional MRI localizer for monocular locations

All participants also underwent an fMRI-based localiser scan to identify voxels in the early visual cortex associated with stimulus input at the monocular locations, used to present the sequences on the separate learning and recognition tests. Participants were informed that four blocks of trials (50 trials/block, randomised) would be presented, and targets would appear at a single monocular location during each block (200 ms ISI). Participants were encouraged to keep their eyes fixated at the center of the placeholder, and their heads were fixed in position with bilateral bracing with soft foam pads, as in all other stages of the experiment. Participants were also instructed to perform the LDT counting task and respond with a value in the same way as during the response period after each block of stimuli. The LDT counting task was used to ensure that participants attended to the stimuli at each single monocular location. Blocks were separated by a 30 s response period to allow a cumulative value on the LDT counting task to be entered.

Behavioural Eye Tracking Control Experiment: Eye movements during dichoptic sequence learning

To assess the role of voluntary (which countermanded the instruction to fixate) and involuntary eye movements, we conducted a separate behavioural experiment in which eye movements were recorded from both eyes using an infrared eye tracker, at a sampling frequency of 1kHZ and 12 bits resolution (Jazz Novo multisensor eye tracker, Ober Consulting; spatial resolution: 0.1°). Participants were asked to maintain central fixation in order to discourage voluntary eye movements. The experimental design was the same as used in the dichoptic fMRI experiment. The eye tracker was calibrated to each participant at the start of the learning and test phases. Visual fixation was assessed by calculating the frequency of eye movements (saccades) outside of central fixation (>1°), using a minimum velocity criterion of 30 degrees per second. Analysis was carried out using Jazz Manager (Ober Consulting).

Direction of saccades and relationship to the two-location perceived sequence and monocular four-location non-conscious sequence: Eye Movement Analyses

The criteria used to mark the onset and offset of conjugate saccades are similar to those used by other studies [S12]. The onset of the saccade was defined as the time when the eye velocity exceeded 5% of the peak saccadic velocity and the offset was defined as the point at which eye velocity dropped below 100/s. Eye movements with latencies longer than 1000 ms along with express eye movements with very short latencies (< 80 ms) were also rejected.

fMRI control experiment: Experimental procedures

Participants

Seventeen undergraduate students were recruited (mean age of 23±2 years; nine female). All participants gave informed written consent to take part in accordance with the terms of approval granted by the local research ethics committee, the principles expressed in the Declaration of Helsinki, and were naive to the purpose of the study. All participants reported normal or corrected-to-normal vision, no history of neurological disease, and no other contraindications for MRI.

Methods

In the control fMRI study, four fixed sequence locations were available to visual awareness and were presented using the same MRI-compatible display system as described below for the main study; hence, dichoptic masking was not used. The experiment was designed to test for commonalities and differences in the learning-related effect, as measured by the high-level contrast between structured and pseudorandom blocks, described in the main text and below, in relation to the analyses of the learning phase. In line with the dichoptic learning protocol, there were three training runs, with an identical arrangement of structured and pseudorandom sequences (see below). The only difference was that learning runs comprised 356 volumes, and each structured and pseudorandom block lasted for 60 seconds. In keeping with to the dichoptic learning protocol, participants were exposed to a repeating 12-element second-order conditional visuospatial sequence displayed at four visible locations (SOC sequences were counterbalanced, and based on the same serial order as described below in the dichoptic learning protocol). Participants were instructed to count the number of trials with a large diameter target in order to ensure that attention was sustained to the visible sequence of stimuli. All parameters of the LDT task were the same as on the dichoptic sequence learning protocol. Observers were also directed to maintain central fixation, and eye movements were monitored during scanning to ensure adherence to the instruction to maintain fixation (XY MRI Oculomotor, Ober Consulting). Infrared reflectance was digitized at 12-bit resolution. Acquisition rate was 250Hz. Minimum resolution in both vertical and horizontal was 0.1 degree. Linearity was assured for ±15°. The eye tracker was calibrated before each run at fixation (central position) and 8 eccentric points. Analyses of eye position showed that participants did not saccade to the target locations of the visible sequence (mean = 2.3, saccades/block of trials). Hence, the learning protocols with and without dichoptic masking were equated in terms excluding eye movements that coincided with the structure of the SOC visuospatial sequence, and manual responses were not performed in relation visuospatial sequence.

Functional and structural MRI data acquisition

Functional MRI series were acquired using a 3.0-Tesla scanner fitted with a 32-channel head coil (Siemens, Verio, Erlangen, Germany) in the main study and fMRI control experiment. The first two runs of the learning phase consisted of 504 volumes, whereas run 3 consisted of 600 volumes due to the additional sequence block. Three hundred volumes were acquired on the recognition test and 250 volumes were acquired on the functional localiser.

Functional volumes consisted of multi-slice T2*-weighted echoplanar images (EPI) with blood oxygenation level dependent (BOLD) contrast. We used the following scanning parameters to achieve whole brain coverage: TR = 2500 ms, TE = 30 ms, 39 coronal slices, 3 mm slice thickness, 0% interslice gap, and FoV = 220 x 220 mm. The resulting voxel size was 3.0 x 3.0 x 3.0 mm. To facilitate anatomical localization and cross-participant alignment, a high-resolution whole-brain structural T1-weighted three-dimensional magnetization-prepared rapid gradient echo (MP-RAGE) scan was acquired for each participant after the EPI imaging (176 sagittal slices TR = 1900 ms, TE = 2.48 ms, FoV= 250 x 250 mm, voxel size = 1.0 x 1.0 x 1.0 mm).

fMRI Image Preprocessing

Image preprocessing and statistical analysis of whole-brain fMRI data were carried out using FEAT (FMRI Expert Analysis Tool; version 5.98), part of the FSL (FMRIB

software library, version 5) [S13]. The Brain Extraction Tool (BET) was used to segment brain matter from non-brain [S14]. The first 6 volumes of each run of the behavioural tasks were discarded to allow for magnetic saturation effects/MR signal stabilization. Image pre-processing involved realignment of EPI images to remove the effects of movement between scans using MCFLIRT [S15], spatial smoothing using a 6-mm full-width-half-maximum Gaussian kernel, pre-whitening using FILM and high-pass filter with a cut-off frequency of 1/100 Hz. Translational movement parameters did not exceed 1 voxel in any direction for any participant or session. Images were registered to the high-resolution structural image (7 degrees of freedom) and then the standard MNI152 template, using affine registration (12 degrees of freedom) [S15]. Statistical analyses were performed in the native image space, with the statistical maps normalized to the standard space prior to higher-level analysis.

fMRI Statistical Analyses

Dichoptic sequence learning phase

The learning data were analyzed using voxel-wise time series analysis within the framework of the General Linear Model (GLM). First-level general linear model analyses for each individual run were performed with the following explanatory variables (EVs) along with their temporal derivatives: sequence (structured) blocks and baseline (unstructured, pseudorandom) blocks, using the FILM module of FSL with local autocorrelation correction [S16]. The design matrix was generated with a synthetic hemodynamic response function modeled as a double gamma function.

Sequence blocks were modeled in chunks of 48 trials (i.e., S_A and S_B) for the purposes of contrast with pseudorandom baseline blocks (48 trials); the value was selected as a multiple of 12-element unit of SOC sequence that allowed the number of trials on sequence blocks to be equated with the baseline blocks. A 30 s interval associated with the response period on the LDT counting task followed each sequence and baseline block. We performed within-subject cross run (fixed-effects) analyses testing for two separate linear contrasts (indicated by the bold text and underlined letter of the 48 trial chunk below) performed on the first-level parameter estimates: one based on corresponding chunks of sequence blocks across the three runs of the learning phase (i.e., run 1 [S_{AB} S_{AB} R S_{AB} R $\underline{\mathbf{S}}_{\underline{\mathbf{A}}B}$] < run 2 [$S_{AB}$ S_{AB} R S_{AB} R $\underline{\mathbf{S}}_{\underline{\mathbf{A}}B}$] < run 3 [S_{AB} S_{AB} R S_{AB} R $\underline{\mathbf{S}}_{\underline{\mathbf{A}}B}$ S_{AB}]); the other examined the reciprocal contrast (i.e., 1 0 -1). Each EV specified the onset and duration of each chunk block. The output of these linear contrasts across the three learning runs (e.g., -1 0 1) was fed into a mixed-effects model for whole-brain group analysis, using FLAME (Local Analysis of Mixed Effect) stage 1 and 2 [S17, S18], testing for consistent learning effects across participants. Group Z (Gaussianized T) statistic images were thresholded using clusters determined by $Z > 2.3$ and a corrected cluster extent significance threshold of $p = 0.05$, using Gaussian Random Field Theory.

It is important to note that because the pseudorandom baseline sequences were visibly different (i.e., at the perceived conscious level) from the repeating structured (second-order conditional) non-conscious sequence, the contrast of BOLD responses between structured and blocks pseudorandom during learning could reflect recognition of the difference in the perceived serial order, rather than sensitivity to the structured non-conscious sequence, per se. Therefore, in order to directly compare the learning effect of the structured sequence relative to the pseudorandom baseline, we used the following mass-univariate approach.

We computed *within-run* statistical maps that were expected to be sensitive to the nature of structured and pseudorandom sequences. Hence, we derived brain maps for the repetition of the structured sequence (i.e. S3>S4: [S1-S2-R1-**S3**-R2-**S4**]) on each of the three training runs. Correspondingly, we derived separate brain maps for the repetition of the pseudorandom sequence during a run (i.e. R1>R2: [S1-S2-**R1**-S3-**R2**-S4]) on each of the three training runs. The respective within-run estimates for the structured and for the pseudorandom sequences were submitted separately to across-run within-subjects fixed effects analyses, testing for linear modulations across the three training runs. Finally, we performed a group-level paired t-test to assess which brain regions were associated with increased training effects in the structured relative to the pseudorandom sequence (and vice versa).

Notably, unlike the recognition test, attention could have acted as an enabling condition during the learning phase, because participants could have allocated attentional resources to the perceived L-R positions in a different manner when encountering the pseudorandom blocks interspersed amongst the repeating non-conscious structured sequence blocks. Therefore, this analysis allows for the direct comparison of the learning effects for structured and pseudorandom sequences, while addressing the confound derived from fact that the sequence types were perceptually different, because structured and pseudorandom sequences were not directly compared at the lower-level of analysis (i.e., within a run). Additional analyses to address this issue are presented in the main text.

Dichoptic non-conscious recognition memory test

The onsets of the old and new retrieval cues were modeled as explanatory variables in the first-level analysis to estimate within-subject differences in BOLD responses. The duration of these EVs corresponded to the duration of each stimulus sequence (retrieval cues) plus the 8 s period assigned for an 'old'/'new' trial. Given that the retrieval cues had a long duration (7.2 seconds), alongside the need to provide exposure to at least two stimuli to enable the first SOC triplet to be recognised, with recognition of additional SOC triplets triggered by further individual stimuli [S8], and masking of the salient information from visual awareness, we elected to include the old/new response period in the model because participants' reflective processes during the old/new decision period may be relevant here to guide non-conscious recognition memory.

Note that two regressors of no interest were also included in the design matrix to control for variation in decision response time for the old/new response period and response period associated with the confidence rating. Two contrasts of parameter estimates were derived from the first-level analyses (old < new sequence: -1 1; old > new 1 -1), which were subsequently submitted to a higher-level mixed effects model testing for consistent effects across participants.

The mass-univariate analyses described above were also implemented on an individual basis using V1 region-of-interest. Individual V1 masks were derived by combining cortical activity maps in a functional localiser of the sequence locations (see below), with an automatic estimate of the location of V1 from cortical folds that was obtained using Freesurfer (http://surfer.nmr.mgh.harvard.edu/), following the method developed by Hinds and colleagues [S19].

Functional MRI localizer for monocular locations

Each of the four monocular locations was modeled as separate EVs in the first-level of the design matrix, along with the response period on the LDT counting task. We used statistical contrasts for the difference in activity between the left and right monocular locations and derived statistical maps of voxels for each individual. Finally, an individual V1 mask was derived from the intersection of voxels that exhibited activity on the functional localiser and the V1 space delineated by Freesurfer.

Analyses of functional connectivity associated with non-conscious recognition memory

In order to assess whether functional connectivity between the hippocampus and V1 was modulated during the recognition test, we used a seed-voxel based approach based on psychophysiological interactions (PPI) to identify signals of interest that would be missed in standard subtraction based analyses. A mask of the hippocampus was drawn from the responsive voxels in the old<new mass-univariate contrast, and this mask was used to define the seed region's time course for the PPI. We then estimated a model for each participant that included psychological EVs for the onsets of old and new trials, EVs for the recognition and confidence periods (as described above for the mass-univariate analysis), and, critically, for the PPI model, a physiological regressor for the time course of the region-of-interest, and psychological x physiological interaction EVs for the PPI. These new regressors were, therefore, added to the previous first-level model for each participant/run. Parameter estimates for old vs. new trials based on the PPI regressors were derived in a similar fashion to the analyses outlined above (e.g., using both fixed-effects analysis across runs followed by mixed-effect analysis across participants), which, here, directly compared the changes in functional coupling associated with training. Given our *a priori* interests in primary visual cortex, this analysis used a region-of-interest approach, with a target occipital mask.

Supplemental Results

Results of the LDT counting task

Mean accuracy in the LDT counting task during the sequence learning phase was 98.2% (S.E.M.= 0.83), and performance did not vary as a function of block type (sequence vs. baseline) ($F_{(1,17)} < 0.1$), or, across the learning phase ($F_{(18,306)} = 1.23$, $p = 0.24$). These data provide evidence of the ability of participants to sustain attention to the onset of targets at the two perceived locations.

Results of post-learning phase awareness questionnaire

Immediately after the dichoptic learning phase, we administered a debriefing questionnaire. Participants exhibited little or no knowledge about the sequence (M rating on the awareness questionnaire = 1.5 [the sequence was "random"], S.E.M.=0.27), and the knowledge expressed was not significantly different from random (=1) ($t_{(17)} = 1.84$, $p>0.05$). These data are in agreement with the results from the LAT (see above, Section 1.1), and demonstrate that *ex post facto* knowledge about even the overall basic structure of the sequence was below subjective threshold.

Results of the signal-detection analyses of the non-conscious (dichoptic) recognition memory test

A receiver-operating-characteristic (ROC) based analysis [S20, S21] was applied to measure type-1 and type-2 sensitivity. In order to conduct the type-1 perceptual sensitivity analyses (i.e., ability to distinguish stimuli), the 'signal' and 'noise' were defined as old and new retrieval cues, respectively. A 'hit' was, therefore, a correct response ('old') to an old trained (six-element sequence) retrieval cue and a 'correct rejection' was a correct response ('new') to a new untrained (six-element sequence) retrieval cue. A 'false alarm' was an incorrect response ('old') to a new retrieval cue and a 'miss' was an incorrect response ('new') to an old retrieval. To obtain the type-1 ROC curves, we plotted the probability of hits as a function of the probability of false alarms for all possible decision criteria. The different points in the ROC curve were obtained by calculating cumulative probabilities for hits and false alarms along a recoded confidence continuum ranging from 3 (i.e., *certain*) to 1 (i.e.. *least certain*) in signal present trials (old retrieval cues), and from 3 (i.e., *certain*) to 1 (i.e., *least certain*) in noise trials (new retrieval cues). On the basis of simple geometry, we computed the area under the ROC curve as a distribution-free measure of the discriminability [S22].

In order to conduct the type-2 meta-d' sensitivity analyses, the area under the ROC curve was calculated by plotting the cumulative probability of correct responses (either hit or correct rejection) and the cumulative probability of incorrect responses (either false alarm or miss) across the different levels of confidence. The area under the ROC curve was estimated as distribution-free measure of metacognitive ability [S23]. Type-1 d' and meta-d' were computed [S24]. Meta-d' is a parametric estimation of type-2 sensitivity predicted from the type-1 signal detection theoretic (SDT) model, computed by fitting a type-1 SDT model to the observed type-2 performance data and estimating the type-2 ROC curves. It corresponds to the efficacy with which the observers' confidence ratings discriminated between their own correct and incorrect stimulus classifications. All data processing and analyses were completed using R (version 3.2.2) [S25] and Matlab (The MathWorks Inc.).

The results are depicted in Figure S3, which includes plots of the area under the type-1 and type-2 curves, including an illustration of the type-2 ROC and also type-1 d' and type-2 meta d' (see below). Table S1 (see above) shows the proportion of 'old'/'new' responses for old(trained) and new(untrained) retrieval cues. Table S2 (see above) also shows the proportion of responses for each confidence rating.

Results of the location awareness test (LAT).

All participants who took part in the fMRI study were administered the LAT on completion of the dichoptic non-conscious recognition memory test. Performance on the LAT was first assessed by calculating a measure of perceptual sensitivity, d' [S26]. Each response was scored as follows: one of the responses (e.g., '1') was treated as signal present and the other response (e.g., '3') as signal absent. Thus, responding with '1' to targets at position 1 was labeled as a, hit, whereas responding with 1 to targets at position 3 was recorded as a, false alarm. The same procedure was applied in the case of 'right' side perceived targets at positions 2 and 4. In this way, we obtained the probability of hits – P(H) – and false alarms – P(FA) – to calculate, d'.

There was no evidence of conscious access to the information that could support accurate identification of the monocular locations, when the proportions of correct responses were analyzed. In particular, participants were at chance (0.5) when identifying the locations of monocular targets that appeared at locations 1 and 3 (i.e., location information in perceived

left targets; Mean (M) proportion correct responses = 0.48, S.E.M. = 0.02; $t_{(17)}$ = -0.63, p = 0.53) and at locations 2 and 4 (i.e., location information in perceived right targets; M = 0.47, S.E.M. = 0.02, $t_{(17)}$ = -1.49, p = 0.15). These results are depicted in Figure S1. Repeated measures t-tests indicated that sensitivity scores for perceived left targets ($t_{(17)}$ = -0.41, p = 0.68) and for perceived right targets ($t_{(17)}$ = 0.58, p = 0.57) did not differ from chance (d' = 0), despite extensive perceptual training and information about the mapping between the four-monocular locations and two perceived locations, before responding on the LAT.

In keeping with standard criteria for the absence of visual awareness, namely d'=0 [S27], the null sensitivity observed in the LAT indicates that observers could not identify the monocular locations that specified the SOC sequence. This was predicted because our experimental protocol provided continuous masking of the monocular sequence locations through binocular fusion. Moreover, these data also broadly align with ceiling performance on the LDT counting task, which provides an independent source of data regarding the stability of binocular fusion inside the scanner, because ceiling performance is dependent on stable fusion [S3, S4]. Finally, the results from the LAT also align with phenomenological reports from the participants who consistently reported in the post-test debriefing and dichoptic calibration sessions that two target locations were experienced in their visual field.

In a revised version of the LAT, inclusion of a confidence rating after each response could be used to assess whether such ratings align with the null sensitivity found on proportion correct and d'. Importantly, if confidence ratings or type-2 sensitivity were diagnostic of item location in the presence of null d', this would arguably be consistent with a process that is dissociable from visual awareness [S28, S29], given that d' was null when probing location information on the LAT. Such a pattern would indicate that non-conscious coding of location information, as well as non-conscious coding of more complex (SOC) associations, are both capable of yielding insight into higher-order non-conscious recognition processes.

In summary, these results suggest that our dichoptic experimental protocol was effective at masking the location information associated with the non-conscious sequence from visual awareness, and, are consistent with our previous behavioural studies [S3, S4].

Results of the eye tracking control experiment

A separate control behavioural experiment was conducted to test whether eye movements were associated with the non-conscious sequence. In the same way as in the main fMRI experiment, participants were instructed to maintain central fixation, and did not perform manual responses during the dichoptic learning and test phases. We examined whether the structure of voluntary and involuntary motor responses (eye movements that violate the instruction to fixate) were correlated with the non-conscious visuospatial sequence specified at the four masked monocular locations or with the visible two-location sequence appearing at the two perceived locations. This question was highly relevant to understanding whether dichoptic sequence learning protocol did, in fact, operate in the absence motor-based learning mechanisms. Notably, the results from the eye movement data cannot exclude a role for responses based on covert reorienting of visuospatial attention, because it remains conceivable that the structure of covert reorienting of visuospatial attention coincided with the perceived or masked visuospatial sequence. Future studies based on analyses of the structure of microsaccades during dichoptic presentation may provide further insight into this issue [S4, S30].

The results demonstrated that participants' maintained visual fixation. Even when involuntary eye movements occurred (despite the instruction to fixate), their direction, and, thus structure, was uncorrelated with the structure of the perceived two-location sequence or with the non-conscious sequence presented at the four monocular locations. The frequency of saccades (i.e., eye movements that violated the instruction to maintain central fixation) during the learning phase did not differ as a function of run or block type (repeated measures ANOVA, p=0.9, and, p=0.89, respectively), and there was no significant interaction between run and block (F<1). These results rule out the possibility that the reported learning effects were caused by differences in eye movements between sequence and baseline blocks. Furthermore, neither the direction of saccades and appearance of stimuli at the four monocular locations, nor, the appearance at the two perceived locations were significant correlated (monocular: r=0.13, p>0.05; perceived: r=0.05, p>0.05). Therefore, the results from this proxy for the allocation of attention, based on the recording eye movements during dichoptic presentation [S31], suggest that sequence learning in our paradigm operated without motor learning based mechanisms, because motor responses were uncorrelated with the structure of the four-location non-conscious or two-location perceived visuospatial sequence.

Supplemental References

S1. Schurger, A. (2009). A very inexpensive MRI-compatible method for dichoptic visual stimulation. J Neuro Methods *177*, 199-202.

S2. Schurger, A., Pereira, F., Treisman, A., and Cohen, J.D. (2010). Reproducibility distinguishes conscious from nonconscious neural representations. Science *327*, 97-99.

S3. Rosenthal, C.R., Kennard, C., and Soto, D. (2010). Visuospatial sequence learning without seeing. PloS One *5*, e11906.

S4. Rosenthal, C.R., Antoniades, C.A., Kennard, C., and Soto, D. (2013). Saccades do not reflect the learning and recognition of an unseen sequence of events. Neuro-Ophthalmol. *37(S1)*, 1–109.

S5. Rosenthal, C.R., Roche-Kelly, E.E., Husain, M., and Kennard, C. (2009). Response-dependent contributions of human primary motor cortex and angular gyrus to manual and perceptual sequence learning. J Neurosci *29*, 15115-15125.

S6. Curran, T. (1997). Effects of aging on implicit sequence learning: Accounting for sequence structure and explicit knowledge. Psychol Res *60*, 24-41.

S7. Shanks, D.R., and Johnstone, T. (1999). Evaluating the relationship between explicit and implicit knowledge in a sequential reaction time task. J Exp Psychol Learn Mem Cogn *25*, 1435-1451.

S8. Shanks, D.R., Channon, S., Wilkinson, L., and Curran, H.V. (2006). Disruption of sequential priming in organic and pharmacological amnesia: a role for the medial temporal lobes in implicit contextual learning. Neuropsychopharmacol *31*, 1768-1776.

S9. Nissen, M.J., and Bullemer, P. (1987). Attentional requirements of learning: evidence from performance measures. Cogn Psychol *19*, 1-32.

S10. Schendan, H.E., Searl, M.M., Melrose, R.J., and Stern, C.E. (2003). An fMRI study of the role of the medial temporal lobe in implicit and explicit sequence learning. Neuron *37*, 1013-1025.

S11. Albouy, G., Stephenich, V., Balteau, E., Vandwalle, G., Deseilles, M., Dang-Vu, T., Darsaud, A., Ruby, P., Luppi, P.-H., Degueldre, C., et al. (2008). Both the hippocampus and striatum are involved in consolidation of motor sequence memory. Neuron *58*, 261-272.
S12. Takagi, M., Frohman, E.M., and Zee, D.S. (1995). Gap-overlap effects on latencies of saccades, vergence and combined vergence-saccades in humans. Vision Res *35*, 3373-3388.
S13. Smith, S.M., Jenkinson, M., Woolrich, M.W., Beckmann, C.F., Behrens, T.E., Johansen-Berg, H., Bannister, P.R., De Luca, M., Drobnjak, I., Flitney, D.E., et al. (2004). Advances in functional and structural MR image analysis and implementation as FSL. Neuroimage *23 Suppl 1*, S208-219.
S14. Smith, S.M. (2002). Fast robust automated brain extraction. Hum Brain Mapp *17*, 143-155.
S15. Jenkinson, M., and Smith, S. (2001). A global optimisation method for robust affine registration of brain images. Med Image Anal *5*, 143-156.
S16. Woolrich, M.W., Ripley, B.D., Brady, J.M., and Smith, S.M. (2001). Temporal autocorrelation in univariate linear modelling of fMRI Data. NeuroImage *14*, 1370-1386.
S17. Woolrich, M.W., Behrens, T.E., Beckmann, C.F., Jenkinson, M., and Smith, S.M. (2004). Multilevel linear modelling for FMRI group analysis using Bayesian inference. Neuroimage *21*, 1732-1747.
S18. Beckmann, C.F., Jenkinson, M., and Smith, S.M. (2003). General multilevel linear modeling for group analysis in FMRI. Neuroimage *20*, 1052-1063.
S19. Hinds, O.P., Rajendran, N., Polimeni, J.R., Augustinack, J.C., Wiggins, G., Wald, L.L., Diana Rosas, H., Potthast, A., Schwartz, E.L., and Fischl, B. (2008). Accurate prediction of V1 location from cortical folds in a surface coordinate system. Neuroimage *39*, 1585-1599.
S20. Egan, J.P. (1975). Signal detection theory and ROC-analysis, (New York: Academic Press).
S21. Green, D.M., and Swets, J.A. (1966). Signal detection theory, (Huntington, New York: Wiley).
S22. Kornbrot, D.E. (2006). Signal detection theory, the approach of choice: model-based and distribution-free measures and evaluation. Percept Psychophys *68*, 393-414.
S23. Fleming, S.M., Weil, R.S., Nagy, Z., Dolan, R.J., and Rees, G. (2010). Relating introspective accuracy to individual differences in brain structure. Science *329*, 1541-1543.
S24. Maniscalco, B., and Lau, H. (2012). A signal detection theoretic approach for estimating metacognitive sensitivity from confidence ratings. Conscious Cogn *21*, 422-430.
S25. Team, R.D.C. (2008). R: A language and environment for statistical computing. (Vienna, Austria: R Foundation for Statistical Computing).
S26. Wickens, C. (1992). Engineering psychology and human performance, (New York: Harper Collins).
S27. Dehaene, S., and Changeux, J.P. (2011). Experimental and theoretical approaches to conscious processing. Neuron *70*, 200-227.
S28. Charles, L., Van Opstal, F., Marti, S., and Dehaene, S. (2013). Distinct brain mechanisms for conscious versus subliminal error detection. Neuroimage *73*, 80-94.
S29. Jachs, B., Blanco, M.J., Grantham-Hill, S., and Soto, D. (2015). On the independence of visual awareness and metacognition: a signal detection theoretic analysis. J Exp Psychol Hum Percept Perform *41*, 269-276.

S30. Yuval-Greenberg, S., Merriam, E.P., and Heeger, D.J. (2014). Spontaneous microsaccades reflect shifts in covert attention. J Neurosci *34*, 13693-13700.
S31. Duc, A.H., Bays, P., and Husain, M. (2008). Eye movements as a probe of attention. Prog Brain Res *171*, 403-411.
