# Supplementary figures and images for: Learning and Recognition of a Non-conscious Sequence of Events in Human Primary Visual Cortex
Source: Curr Biol. 2016 Mar 21;26(6):834–41. doi: 10.1016/j.cub.2016.01.040 (PMC4819512; doi:10.1016/j.cub.2016.01.040)

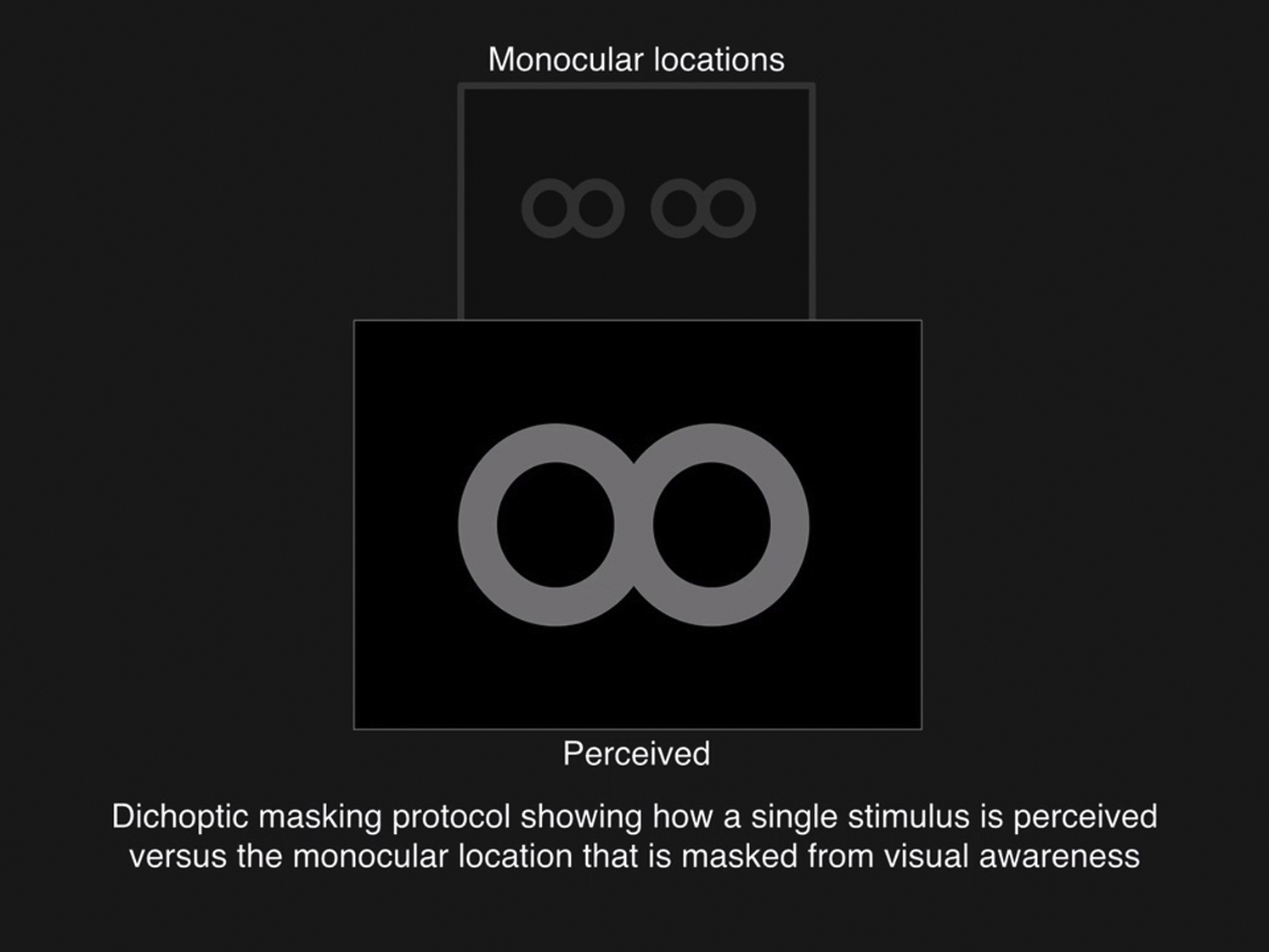

Supplement: Movie S1. Dichoptic Presentation Protocol Used During Learning and Test Phases, the Location Awareness Test, and the Supplemental Eye Movement Control Experiment, Related to Figure 1 — The movie shows how monocular locations were masked from visual awareness by the use of dichoptic viewing through a prism-based stereoscope inside the bore of the MRI scanner. Head position and eye position were fixed throughout all fMRI data acquisition stages. The foreground two-location placeholder corresponds to the image perceived when the four locations were viewed through the prism-based stereoscope. First, a 12-element second-order sequence was repeatedly presented across four monocular locations on a computer screen. Note the perceived (two-position) binocular sequence contains no eye-of-origin information from the four monocular stimulus locations in the non-conscious sequence (separately verified using data analyzed using signal detection theory). Stimuli presented in locations 1 and 3 were perceived on the left, whereas stimuli presented in locations 2 and 4 were perceived on the right. Second, an example six-item “old” recognition retrieval cue is shown (i.e., in the format of presentation used on the dichoptic recognition test) and followed by an illustrative parallel presentation of six-item “old” and “new” recognition retrieval cues to depict how the perceived serial order was matched between these two types of retrieval cue. As the illustration of the matching of old and new retrieval cues depicts, the perceived serial order associated with each old and new retrieval cue were equated so that recognition memory could be based only on sensitivity to serial order of the old/new non-conscious sequence (specified across the four monocular positions). In all example trial sequences, the rate of stimulus presentation is slowed for illustrative purposes (see Supplemental Experimental Procedures for the SOA values used in the experiments). [file mmc2.jpg]
