# Supplementary material for: Learning and Recognition of a Non-conscious Sequence of Events in Human Primary Visual Cortex
Source: Curr Biol. 2016 Mar 21;26(6):834–41. doi: 10.1016/j.cub.2016.01.040 (PMC4819512; doi:10.1016/j.cub.2016.01.040)
Supplement: Document S2. Article plus Supplemental Information [file mmc3.pdf]

Current Biology

Report

Learning and Recognition of a Non-conscious Sequence of Events in Human Primary Visual Cortex

Highlights

- Sequence learning and recognition memory can operate without visual awareness
- V1, hippocampus, and basal ganglia support learning of a non-conscious sequence
- Old/new status of non-conscious recognition probes modulates V1-hippocampal coupling
- V1 activity predicts non-conscious recognition memory performance

Authors

Clive R. Rosenthal,
Samantha K. Andrews,
Chrystalina A. Antoniades,
Christopher Kennard, David Soto

Correspondence

clive.rosenthal@clneuro.ox.ac.uk
(C.R.R.),
d.soto@bcbl.eu (D.S.)

In Brief

Rosenthal et al. demonstrate that responses in human primary visual cortex (V1) support perceptual learning and recognition of a complex and non-conscious visuospatial sequence, and predict high-level, non-conscious recognition memory performance. These results elaborate on V1's established role in simple, low-level perceptual learning.

CrossMark
Rosenthal et al., 2016, Current Biology *26*, 834–841
March 21, 2016 ©2016 The Authors
http://dx.doi.org/10.1016/j.cub.2016.01.040

CellPress

CellPress

Current Biology
Report

Learning and Recognition of a Non-conscious Sequence of Events in Human Primary Visual Cortex

Clive R. Rosenthal,[1,*] Samantha K. Andrews,[2] Chrystalina A. Antoniades,[1] Christopher Kennard,[1] and David Soto[3,4,*]
[1]Nuffield Department of Clinical Neurosciences, University of Oxford, Oxford OX3 9DU, England, UK
[2]Department of Experimental Psychology, University of Oxford, Oxford OX1 3UD, England, UK
[3]Basque Center on Cognition, Brain and Language, Paseo Mikeletegi, 20009 San Sebastian - Donostia, Spain
[4]Ikerbasque, Basque Foundation for Science, 48013 Bilbao, Spain
*Correspondence: clive.rosenthal@clneuro.ox.ac.uk (C.R.R.), d.soto@bcbl.eu (D.S.)
http://dx.doi.org/10.1016/j.cub.2016.01.040
This is an open access article under the CC BY license (http://creativecommons.org/licenses/by/4.0/).

SUMMARY

Human primary visual cortex (V1) has long been associated with learning simple low-level visual discriminations [1] and is classically considered outside of neural systems that support high-level cognitive behavior in contexts that differ from the original conditions of learning, such as recognition memory [2, 3]. Here, we used a novel fMRI-based dichoptic masking protocol—designed to induce activity in V1, without modulation from visual awareness—to test whether human V1 is implicated in human observers rapidly learning and then later (15–20 min) recognizing a non-conscious and complex (second-order) visuospatial sequence. Learning was associated with a change in V1 activity, as part of a temporo-occipital and basal ganglia network, which is at variance with the cortico-cerebellar network identified in prior studies of "implicit" sequence learning that involved motor responses and visible stimuli (e.g., [4]). Recognition memory was associated with V1 activity, as part of a temporo-occipital network involving the hippocampus, under conditions that were not imputable to mechanisms associated with conscious retrieval. Notably, the V1 responses during learning and recognition separately predicted non-conscious recognition memory, and functional coupling between V1 and the hippocampus was enhanced for old retrieval cues. The results provide a basis for novel hypotheses about the signals that can drive recognition memory, because these data (1) identify human V1 with a memory network that can code complex associative serial visuospatial information and support later non-conscious recognition memory-guided behavior (cf. [5]) and (2) align with mouse models of experience-dependent V1 plasticity in learning and memory [6].

RESULTS

Recent evidence has identified mouse primary visual cortex (V1) with coding simple serial associations and timing information, when learning a repeating sequence of visible gratings [7], and with a form of recognition memory that is selective for the orientation of visible gratings [6]. In humans, a frontal-occipital negative-going event-related potential (ERP) signal has been identified with a guess response that is associated with the recognition of single visible items [8]. It is thus conceivable that experience-dependent changes in human V1 may be relevant for later recognition memory-guided behavior, but critically, this needs to be established outside of the general modulation in V1 associated with visual awareness [9].

We combined fMRI with a novel dichoptic masking protocol, involving separate learning and recognition phases associated with the presentation of a complex and non-conscious visuospatial sequence of targets appearing across four monocular locations. Figure 1 illustrates the protocol (see Figure 1 legend for details; see also Movie S1 and Supplemental Experimental Procedures for further details).

This visual presentation protocol was expected to be associated with V1 activity because monocular inputs are retained in early visual cortex [10] and dichoptic masking has been identified with responses in V1 [11]. During the learning phase, we ensured that the observers sustained their attention to single targets that appeared on each trial by asking them to count the number of trials with a large-diameter target (LDT; targets could be of either standard or large size—see Supplemental Experimental Procedures). Performance on the LDT counting task was at ceiling (see Supplemental Results). The LDT counting task was also expected to drive V1 activity, because V1 codes for magnitude differences between stimuli [12].

An unexpected non-conscious recognition memory test followed 15–20 min after the learning phase (see Figure 1 and its accompanying legend for a description of the recognition procedure and the features of the retrieval cues). On each trial of the recognition test, observers were asked to discriminate whether the retrieval cue was drawn from the old (trained) sequence or from a new sequence and to rate the confidence in their response on a six-point scale (1–3, "old"; 4–6, "new"; see Figure 1). Crucially, old and new retrieval cues were perceived in the same serial order due to dichoptic presentation. As can be seen in Figure 1, the sequence of locations that distinguished old (e.g., 3-4-1-2-4-3) from new retrieval cues (e.g., 1-4-3-2-4-1) was confined to the monocular level. For the above examples, both old and new retrieval cues were perceived as L-R-L-R-R-L (see Table S3 and Figure 1). Therefore, serial order information that distinguished old and

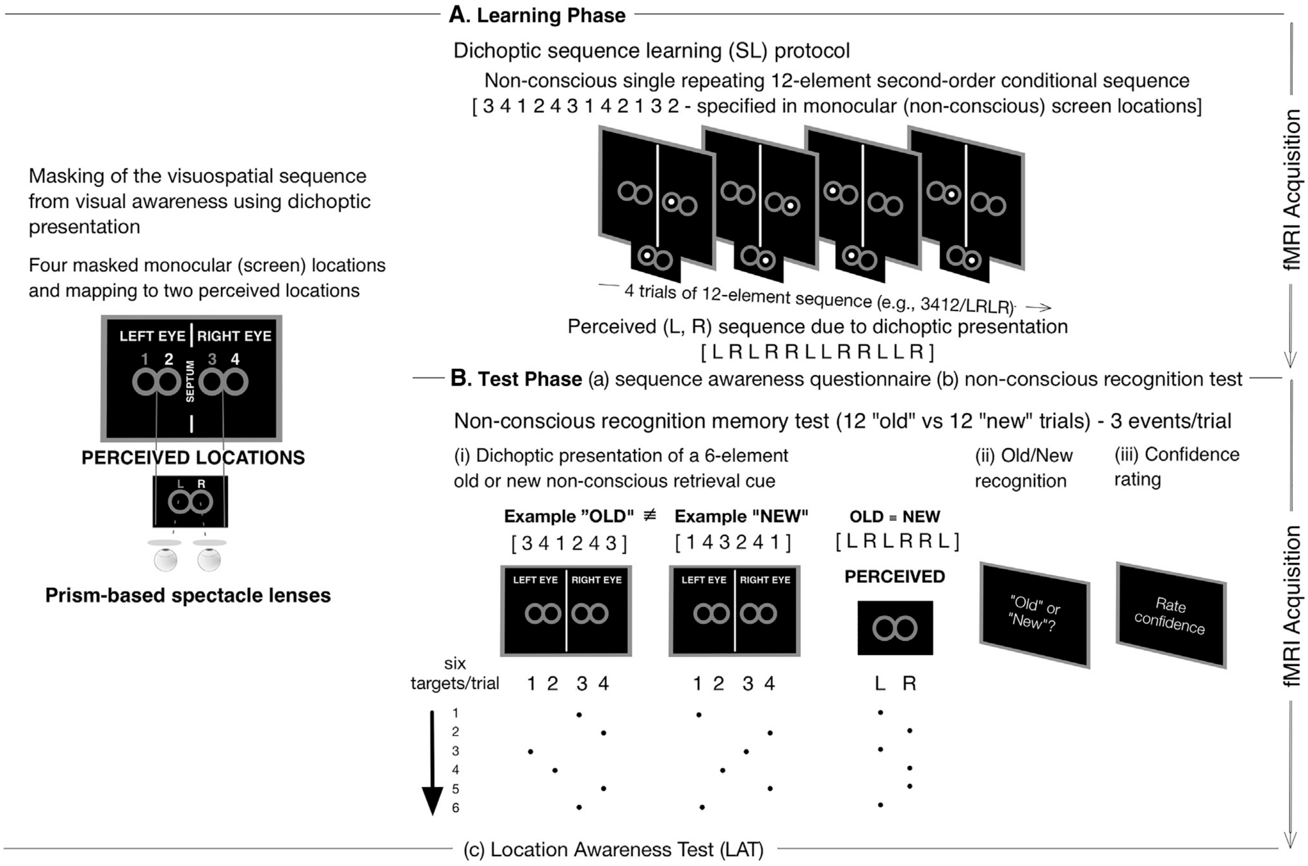

Figure 1. Experimental Procedure and Stimuli

(A) Learning phase. First, in a learning phase that lasted approximately 1 hr 15 min, a visuospatial sequence based on a 12-element second-order conditional (SOC) rule specified across four monocular locations (1-2-3-4) was presented repeatedly to induce discontinuous relational coding of serial interocular and monocular associations. Each target was presented for 2000 ms at the center of one of four monocular locations circumscribed by two horizontally aligned and isoluminant figures-of-eight (read from left to right; placeholders 1 and 2 were in the left figure-of-eight and stimulated the left eye whereas placeholders 3 and 4 were in the right figure-of-eight and stimulated the right eye). The four monocular locations were continuously masked from visual awareness by dichoptic presentation: a septum inside the bore of the scanner and spectacles fitted with prism lenses were worn by observers to create two independent visual channels for each eye, which led to the binocular fusion of the four monocular locations into two perceived locations, circumscribed within a single, fused, centrally positioned, and horizontally aligned figure-of-eight. Monocular locations 1 [left eye-of-origin channel] and 3 [right eye-of-origin channel] were perceived in the left placeholder (L), whereas monocular locations 2 [left eye-of-origin channel] and 4 [right eye-of-origin channel] were perceived in the right placeholder (R). This method of dichoptic presentation eliminated crosstalk between each eye-of-origin, enabled binocular fusion to be sustained for long periods, and provided continuous masking of the four monocular target locations in which the second-order conditional sequence was embedded. Observers maintained a fixed head position and were encouraged to attend to the stimuli at the two perceived locations by the requirement to count the number of perceived large-diameter targets across each block of trials.

(B) Test phase (three discrete stages). (a) Sequence awareness questionnaire: observers responded immediately after the learning phase to indicate whether they had detected any regularities in the stimulus presentation. (b) Non-conscious recognition memory test (for behavioral results, see Figures S2 and S3 and Tables S1 and S2): sequence knowledge was assessed on the unexpected recognition test (implemented using dichoptic presentation) administered 15–20 min after the learning phase. Each trial of the recognition test (b) involved three phases: (i) observers were presented with a retrieval cue comprised of a six-element sequence of targets (7.2 s), which was drawn from six-element segments of either the old (trained) 12-element SOC sequence or a new (untrained) 12-element SOC sequence. 12 old and 12 new recognition trials were presented in a random order. Notably, the perceived serial order of old and new retrieval cues was equated, as were all other stimulus dimensions and structural properties that would otherwise serve as a basis for a perceived difference that could enable discrimination (i.e., the frequency with which each location occurred, transitions between the four locations, reversals, and laterality) (see also Table S3). (ii) Observers were asked to perform an old/new recognition-based discrimination response during a limited time window of 8 s. (iii) Observers were required to rate their confidence in the old or new response (8 s) on a six-point scale (if "old"/trained, assign a value ranging in confidence between 1 [certain] and 3 [least certain], or, if "new"/untrained, assign a value between 4 [least certain] and 6 [certain]). (c) Location awareness test (LAT) (for results, see Figure S1): the LAT was administered inside the scanner to test the efficacy with which the four monocular locations were masked from visual awareness. Each trial of the LAT presented observers with a target at one of the four monocular positions. Participants were instructed on the mapping between each monocular stimulus and the corresponding perceived binocular location and were then asked to discriminate between monocular locations 1 and 3 for "left" perceived targets or between monocular locations 2 and 4 for "right" perceived targets. Targets remained on screen until a manual response was entered on the response pad and were separated by a 200 ms inter-stimulus interval.

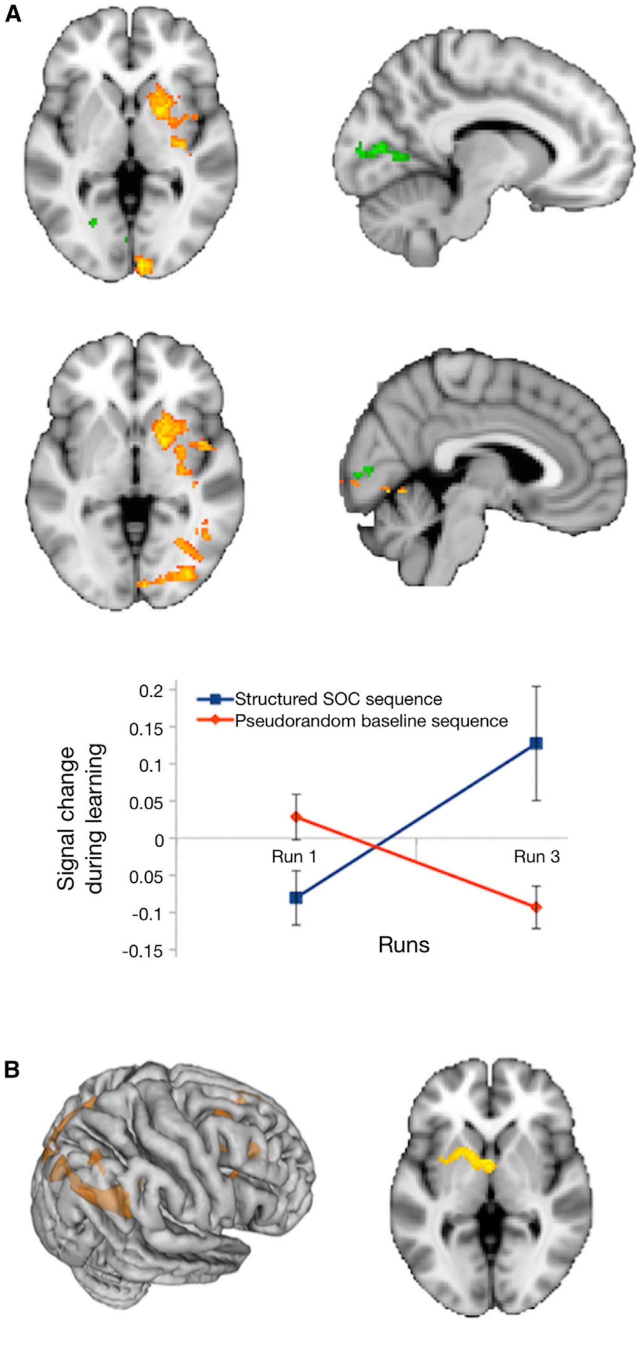

Figure 2. Changes in BOLD Activity Associated with Non-conscious Learning and Learning a Visible Sequence

(A) Brain regions exhibiting linear changes in BOLD signal for structured and pseudorandom sequences across the dichoptic learning phase ($Z > 2.3$, $p < 0.05$, whole-brain corrected; [14]), rendered onto axial and sagittal views. The yellow clusters depict the brain areas in which the learning effects across runs were higher for the structured sequence blocks relative to the pseudorandom blocks. We computed within-run statistical contrasts that were sensitive to the nature of structured (S) and pseudorandom (R) sequences, namely, for the repetition of the structured sequence (i.e., S3 > S4: [S1-S2-R1-S3-R2-S4]) on each of the three training runs. Correspondingly, for each run of the learning phase, a contrast test for the attenuation of the neural response with the repetition of structured sequences (e.g., S3 > S4) was derived (and likewise for the pseudorandom sequences, e.g., R1 > R2). The respective within-run estimates for the structured and for the pseudorandom sequences were submitted separately to across-run within-subject fixed-effects analyses, testing for linear modulations across the three training runs. Finally, we performed a group-level paired t test to assess which brain regions were associated with increased training effects in the structured relative to the pseudorandom sequence (and vice versa). The graph of signal change during learning depicts the linear estimates of these neural repetition effects across fMRI runs (average of all clusters of activity depicted in yellow; error bars correspond to SEM). Hence, the linear increase for structured blocks (in blue) reflects increased repetition attenuation with learning for the structured sequence, but this was not the case for the pseudorandom sequence (in red). The green clusters show brain regions exhibiting a linear change in BOLD signal for the structured blocks only across the training runs ($Z > 2.3$, $p < 0.05$, whole-brain corrected). Both yellow and green designated clusters include the intracalcarine cortex of primary visual cortex. Group-based fMRI analyses report anatomical regions based on Harvard-Oxford Probabilistic Atlas, as part of FSL [13].

(B) BOLD activity map associated with learning a visible second-order conditional visuospatial sequence ($Z > 2.3$, $p < 0.05$, whole-brain corrected), obtained when using an equivalent linear contrast to that described above on the dichoptic learning protocol (i.e., comparing the linear trends for structured and pseudorandom sequences in the control fMRI study). Response changes were observed in the angular gyrus (MNI 58 −56 38, Z = 3.91), precuneus (−4 −78 40, Z = 3.89), middle frontal gyrus (24 12 44, Z = 3.93), and superior frontal gyrus (12 28 58, Z = 3.86). Activity was also found in subcortical foci, including mediodorsal and ventral anterior thalamus (bilateral, 2 −14 12, Z = 4.2), extending into the left putamen (−18 8 −2, Z = 3.04). We found no areas exhibiting a linear change in BOLD signal for the structured blocks across the training runs.

new retrieval cues was not available to visual awareness (as confirmed by psychophysical tests reported below).

Before presenting the neuroimaging data, we report the results from behavioral control experiments demonstrating that (1) the participants did not have conscious access to the four (monocular) locations (see Figure S1 and Supplemental Results) and (2) sequence learning operated independently of motor-based mechanisms, i.e., did not involve somatomotor responses and eye movements that would generate stimulus-motor response bindings coinciding with the structure of the visuospatial sequence (cf. [4, 13]). Observers' inability to identify the monocular locations was consistent with a lack of knowledge, even about the overall basic sequence structure (as assessed on a post-learning awareness questionnaire; see Supplemental Results).

Neural Substrates of Sequence Learning

In line with prior studies of sequence learning, whole-brain analyses tested for linear effects of exposure to the non-conscious structured second-order conditional (SOC)-based sequence by comparing it against a pseudorandom baseline sequence. Estimates were derived separately for structured and pseudorandom blocks, and these were compared at a higher level of analysis (see Figure 2A legend and Supplemental Information). Activity was observed in a set of regions that included right putamen and pallidum (Montreal Neurological Institute [MNI] 20 4 0, Z = 3.62), right insula (40 −2 −14, Z = 3.59), inferior temporal gyrus (46 −54 −8, Z = 3.78), hippocampus (30 −20 −12, Z = 2.59), lateral occipital cortex (34 −82 −2, Z = 3.39), fusiform gyrus (30 −66 −6, Z = 3.61), lingual gyrus (24 −60 −8, Z = 3.56), and occipital pole (8 −96 0, Z = 3.33), which notably included the intracalcarine cortex of primary visual cortex (6 −88 −2, Z = 3.09). Figure 2A depicts the brain activity maps (in yellow).

Learning was also examined by testing linear effects of exposure to the structured sequence alone (Figure 2A, indicated in green), which again showed activity in the intracalcarine sulcus (peak MNI −18 −64 4, Z = 3.3), extending into the lingual gyrus and precuneus. Activity in V1 is thus unlikely to reflect relative differences in novelty of the pseudorandom versus structured sequences within a run, and, as we discuss later, the change in V1 activity predicted behavior on the non-conscious recognition test, which could not be solved on the basis of relative novelty.

Unlike the non-conscious recognition test, where old and new retrieval cues were perceived in the same serial order, there was a visible difference in perceived L-R serial order between structured and pseudorandom blocks during the learning phase (see Supplemental Experimental Procedures), and not all low-level structural properties were equated across the two sequence types. In line with previous studies [15], the serial order of the visual targets (L-R) on unstructured baseline blocks was pseudorandom to facilitate learning of SOC structured sequence. This may have modulated how attention was allocated during learning and hence could account for the observed learning activity in V1. Therefore, a separate control fMRI study was conducted to examine the learning of a SOC sequence under conditions where the four locations were available to visual awareness. All other aspects of the control fMRI study were equated with the dichoptic learning protocol, including the LDT counting task, and learning without motor responses (see Supplemental Experimental Procedures section "fMRI control experiment: Experimental procedures"). Learning the visible sequence was associated with a right fronto-parietal network, which also included subcortical foci in thalamus and left putamen (Figure 2B). These regions were used as a mask to test for learning-related activity in the dichoptic learning protocol. Only the left putamen (−24 10 −2, Z = 3.76, corrected) was associated with learning-related activity under dichoptic masking. Conversely, when the regions that showed learning-related activity during dichoptic masking of the sequence (i.e., primary visual, occipito-temporal cortex, hippocampus, and basal ganglia) were used as a mask to test for learning-related activity associated with the visible sequence, activity was confined to foci in the right putamen (26 4 −10, Z > 2.3, corrected). Again, there was no evidence of additional overlap. Finally, an unpaired t test was performed to compare the parameter estimates for the learning effects associated with conscious visible sequences with those in the dichoptic masking experiment. The results showed that learning-related activity in superior frontal and prefrontal areas was greater with visible than with the masked sequences.

Together, these results indicate that learning-related activity in the dichoptic masking protocol is unlikely to have been driven by perceptual differences between structured and pseudorandom sequences, sensory adaptation, or other non-specific learning effects, because these factors were equated in both fMRI studies, yet the learning-related brain networks did not map across the learning of conscious visible and non-conscious sequences. We present additional information from the non-conscious recognition test below in support of this interpretation.

Behavioral Evidence for Non-conscious Recognition Memory

Non-conscious recognition memory was first examined using a contrast based on confidence ratings associated with all old and all new stimuli (retrieval cues). Although the proportion of correct "old"/"new" discriminations was at chance (mean = 0.51 [SEM = 0.01]; $t_{(17)} = 0.68$, $p > 0.05$), the memory ratings associated with all "old" and all "new" retrieval cues were significantly different (mean "old" = 2.64 [SEM = 0.04], mean "new" = 3.16 [SEM = 0.07]; $t_{(17)} = -7.95$, $p < 0.001$; Figure S2), indicating recognition of the non-conscious sequence. This result is in keeping with recent behavioral evidence of recognition memory without visual awareness [16, 17] and aligns with other behavioral evidence that has examined the learning of non-conscious first-order sequences [18].

Additional analyses were based on receiver operating characteristics (ROC) of type 1 performance (i.e., sensitivity to "old"/"new" retrieval cues) and type 2 performance (i.e., how memory confidence relates to "old"/"new" discriminations), and also type 1 d′ and type 2 meta-d′ [19] (i.e., the efficacy with which observers' confidence ratings discriminated between their own correct and incorrect memory decisions). Type 1 sensitivity for "old"/"new" discrimination was at chance (i.e., for sensitivity based on the area under the type 1 ROC: $t_{(17)} = 1.45$, $p = 0.17$, mean = 0.52, SEM = 0.01, chance = 0.5; for type 1 d′: $t_{(17)} = 0.59$, $p = 0.57$, mean = 0.03, SEM = 0.05, chance = 0), whereas type 2 sensitivity was significantly above chance (for sensitivity based on the area under the type 2 ROC: $t_{(17)} = 5.79$, $p < 0.001$, mean = 0.63, SEM = 0.02, chance = 0.5; for type 2 meta-d′: $t_{(17)} = 4.90$, $p < 0.001$, mean = 0.9, SEM = 0.18, chance = 0) (see Figure S3, Supplemental Results, and Tables S1 and S2). These results are consistent with recent evidence indicating that metacognitive processes (e.g., related to perceptual decision making or memory) can be successfully deployed even when type 1 sensitivity is null [20, 21], i.e., under conditions that are independent of visual awareness [22]. The current study and other recent studies set precedents that require additional experimental work to examine the relationship between visual awareness and higher-order cognition.

There was no significant difference in the manual response times (RTs) to "old" and "new" retrieval cues (mean RTs, 2018 ms versus 2119 ms, respectively; $t_{(17)} = 0.86$, $p = 0.40$). Therefore, any increase in perceptual fluency (i.e., the perceived speed of processing an item), induced by prior exposure, did not lead to faster identification times (response priming), which suggests that response fluency did not serve as a basis on which to recognize prior occurrence (cf. [23]). Notably, prior studies have reported RT differences between old and new retrieval cues based on visible sequences (e.g., [24]). The absence of such priming effects in the current study may be because motor-based responses were not performed during learning.

Relevance of Learning-Related Activity for Subsequent Non-conscious Recognition Memory

The relevance of learning-related activity for knowledge of the trained SOC sequence was examined using a robust multiple regression analysis based on the magnitude of the corrected blood-oxygen-level-dependent (BOLD) signal change during learning within the calcarine sulcus and six other functional

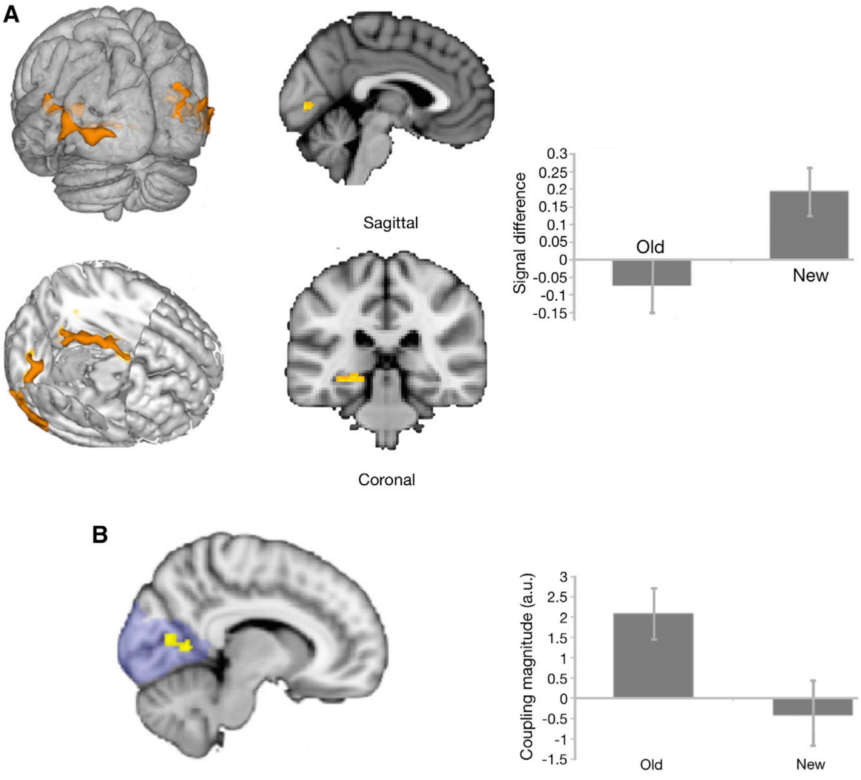

Figure 3. BOLD Responses and Functional Connectivity Associated with Non-conscious Recognition Memory

(A) BOLD responses associated with non-conscious recognition memory (for behavioral results, see Figures S2 and S3 and Tables S1 and S2). Left: brain regions showing BOLD activity for the contrast between old < new sequences ($Z \geq 2.3$, $p < 0.05$, whole-brain corrected). Right: plot of the signal difference in these regions associated with recognition of the non-conscious sequence. Old and new retrieval cues differed only in terms of the monocular target sequence, whereas the perceived serial order associated with the old and new retrieval cues was the same. As an example, the perceived serial order of a non-conscious old cue specified across monocular (1, 2, 3, 4) and eye-of-origin ([L] = left; [R] = right) locations, 3[L]-4[R]-1[L]-2[R]-4[R]-3[L], was matched to a non-conscious new cue specified across monocular and eye-of-origin locations, 1[L]-4[R]-3[L]-2[R]-4[R]-1[L] (see also Table S3). Error bars correspond to SEM.

(B) Results from a psychophysiological interaction-based analysis that examined functional connectivity associated with a hippocampal-based seed voxel drawn from the responsive voxels in the non-conscious old < new recognition memory-based contrast. Functional coupling between the hippocampus and the intra-calcarine cortex (V1, indicated in yellow) was modulated as a function of whether the retrieval cue on the recognition test was old or new ($Z > 2.3$, $p < 0.05$, corrected for the occipital mask in blue shading). The graph on the right shows that the magnitude of the functional coupling was higher for old relative to new retrieval cues. Error bars correspond to SEM.

regions-of-interest (ROIs) (hippocampus, inferior temporal sulcus, insula, putamen, fusiform cortex, and lingual gyrus). Each was entered as a variable in a multiple linear regression using Huber's method of correction for outliers against the magnitude of non-conscious recognition memory (old minus new mean confidence ratings). Learning-related activity within the calcarine sulcus predicted behavior on the non-conscious recognition test ($\beta = 0.35$, $p < 0.05$), whereas the activity estimates from the six other ROIs were not significant predictors of non-conscious recognition memory (all p's > 0.21). If learning-related activity in V1 had merely reflected low-level sensory adaptation effects, low-level structural or relative novelty differences, and/or perceived differences in serial order between the structured and pseudorandom sequences, then the V1 signal change during learning should not have predicted recognition memory, because structural and perceived differences were equated between old and new retrieval cues used on the non-conscious recognition test (Table S3).

Neural Substrates of Non-conscious Recognition Memory

First, we assessed BOLD activity for the old < new contrast using a whole-brain mass-univariate analysis. Non-conscious recognition-related activity was observed in a visual cluster with a peak in the lingual gyrus (MNI −20 −50 −4, Z = 3.7), which extended into the intra-calcarine sulcus in V1 (−4 −80 2, Z = 2.79). Left and right lateral occipital cortex (LOC) also exhibited activity, with peaks in left inferior LOC (−52 −76 −6, Z = 3.64) and right superior LOC (28 −88 26, Z = 4.39). Notably, the old < new effect was also found in the left hippocampus (−22 −38 0, Z = 3.49). Figure 3A depicts the brain activity maps. No significant brain changes were found in the old > new contrast.

Second, we performed the same analyses using functional ROIs derived from the intracalcarine cluster observed during the learning phase, with voxelwise correction for multiple comparisons ($p < 0.05$). Notably, learning and non-conscious recognition-related activity overlapped in the intracalcarine cortex (MNI −0 −80 2, Z = 3.17 and −14 −68 8, Z = 3.29). This overlap suggests that V1 activity can occur even when there are no low-level structural differences between the sequences that form the basis of the contrast, as compared to the structured versus pseudorandom block-based linear contrast in the learning phase.

Third, following previous studies that identified the conscious recall of visible grating pairs with hippocampal-V1 coupling [25] and coupling between the hippocampus and early visual cortex with implicit statistical learning of visible stimuli [26], we tested for functional connectivity between hippocampus and V1 during non-conscious recognition by means of psychophysiological interaction (PPI) analysis. A mask of the hippocampus was drawn from the responsive voxels in the old < new contrast described above and used to define the seed region's time course. Given our a priori interest in V1, this analysis used a region-of-interest approach with an occipital mask. Figure 3B illustrates the PPI results. A significant cluster was found in left V1 (MNI 10 −70 12)

that, critically, coincided with the intra-calcarine cortex ($Z > 2.3$, $p < 0.05$ corrected for the occipital mask). This V1 cluster exhibited increased functional coupling with the hippocampus during the presentation of old relative to new retrieval cues. The same result was observed in the adjacent intra-calcarine area of the right hemisphere ($Z > 2$, $p < 0.05$, corrected). Individual measures of functional connectivity did not correlate with non-conscious recognition memory behavior ($p > 0.05$).

Finally, the link between human V1 and calcarine sulcus is well described [27], and activity in the intracalcarine cortex and its vicinity coincided with probabilistic V1. Nonetheless, given our a priori interest in V1, a functional localizer was administered in a separate fMRI scan to map regions in early visual cortex that were associated with visual stimulation at the monocular locations. V1 was automatically defined on each individual structural scan by applying the method developed by Hinds and colleagues [28]. This method is implemented in Freesurfer [29], in order to predict the location of the stria of Gennari—an anatomical marker of primary visual cortex—with reference to cortical surface topology, which is demonstrated to be as accurate as a retinotopic mapping [30]. Individual masks with a probability of 0.99 of occurring in V1 were obtained on each observer to mitigate uncertainties about architectonic borders. Individual masks were then derived for the intersection of active voxels in visual cortex seen on the localizer and the V1 area derived from Freesurfer. These masks were used to extract individual parameter estimates for the old < new contrast. A one-sample t test showed that the old < new recognition effect was significantly different from chance ($t_{(17)} = 2.14$, $p < 0.05$).

Behavioral Significance of Activity Associated with Non-conscious Recognition Memory

This issue was examined by using the same robust multiple-regression-based method described earlier, but with recognition fMRI-based ROIs in the calcarine sulcus, hippocampus, and right occipital cortex. The difference in activity within the calcarine sulcus predicted behavior in the non-conscious recognition test ($\beta = 0.31$, $p < 0.05$). Likewise, activity in the right occipital cortex also predicted behavior in the non-conscious recognition test, with a negative association ($\beta = -0.47$, $p < 0.05$), whereas the activity estimate from the hippocampus was not a significant predictor ($p = 0.32$). These results suggest that activity in V1 and the occipital cortex both predicted non-conscious recognition memory but that only the magnitude of the old < new response in V1 was linked with improved non-conscious recognition memory. One interpretation of these associations is that non-conscious recognition processes may operate not only by local signal differences in V1 activity but also by concurrent inhibition in connected occipital structures that respond to the monocular stimuli. Push-pull processes of this type may optimize discrimination processes in the service of non-conscious recognition memory, but definitive hypotheses about possible gating mechanisms involving regional activity within occipital cortex associated with non-conscious recognition memory await further investigation.

DISCUSSION

Learning-related activity occurred in cortical and subcortical regions, including primary visual cortex, as part of a temporo-occipital and basal ganglia network. In contrast to the much-studied serial reaction time task used to investigate sequence learning [31], observers did not direct motor responses or motor attention to the visual stimuli. Therefore, activity reflected complex learning in the absence of mechanisms related to motor responding, and the overlap with several regions identified in visuomotor sequence learning [4, 15] is thereby consistent with common mechanisms of perceptual and motor learning [32]. By contrast, V1 activity has not been seen in prior investigations of explicit (conscious) and implicit (non-conscious) human visuomotor sequence learning of visible stimuli, nor was it observed in our control fMRI learning study based on a visible SOC sequence. V1 has however been implicated with coding simple serial associations and timing information in an experimental animal mouse model, when studied using stimuli (visible gratings) designed to induce activity in V1 [7].

Unlike later visual cortical areas such as the perirhinal and infero-temporal cortex, there is little experimental data to implicate V1 in recognition memory. Most notably, recent work in a mouse model has linked V1 with a form of recognition memory based on discriminations between visible old and novel oriented gratings [6, 33]. We extend these observations by showing that early visual cortices in humans, including V1, a region implicated in perception and perceptual learning of low-level features [34], is associated with learning and subsequent non-conscious recognition memory of a repeating complex visuospatial sequence. Importantly, by broadening the scope of investigation to high-level memory-guided behavior, we have demonstrated that experience-dependent changes in V1 can (1) occur even when the spatial location of targets is masked from visual awareness, (2) arise over an hour rather than several days (cf. [1]), (3) operate in the absence of reward or punishment [35], and (4) generate a signal that predicts behavior (i.e., recognition confidence) outside of the original conditions of (perceptual) learning (cf. [36]).

Isolating non-conscious processes in recognition memory has proved difficult to achieve. One potential reason is that previous behavioral paradigms have administered visible stimuli at encoding and as retrieval cues ([8, 37]; but see [16]). The key advantage of our protocol is that activity in visual cortex and the hippocampus were not mediated by modulation due to conscious perceptual expectations (related to the four monocular locations) [38], the reinstatement of episodic information [39], or other old/new differences in processes—such as evaluation and decision making—that operate "downstream" of conscious retrieval. Furthermore, the non-conscious recognition effect seen here is distinguished from other non-conscious forms of memory, such as repetition priming, because repetition-related occipital fluency effects (1) are often unrelated to behavior [40], (2) if present, only prime a particular response such as identification [41], rather than supporting behavior outside of the original study context (i.e., discrimination based on old/new recognition confidence ratings), and (3) involve behavioral phenomena that are considered to be short lived [42], rather than operating 15–20 min after initial learning, as in the current study.

The enhanced functional coupling observed between V1 and the hippocampus during non-conscious recognition is noteworthy (1) given evidence from experimental animal models

that implicate the entire ventral visual-to-hippocampal stream in memory for visible items [43] and (2) in light of data in humans wherein hippocampal activity has been aligned with (conscious) memory strength rather than recollection and familiarity [44] and has been shown to be sensitive to the distinction between (visible) old versus novel stimuli [45]. Unlike in previous studies, the hippocampal activity seen here was not attributable to a general novelty signal, a difference in familiarity, or conscious memory strength (cf. [46, 47]), because the new retrieval cues were not based on novel stimuli with respect to the learning phase (cf. [16]), and the serial order of the (binocular) perceived two-location old and new retrieval cues was equally familiar. Old and new retrieval cues were distinguished only in terms of their unique non-conscious serial order, specified at the masked four monocular locations. More broadly, the observed hippocampal activity is notionally consistent with the proposal that relational complexity, rather than mechanisms associated with conscious perception, modulates hippocampal engagement [48].

In summary, our results identify experience-related plasticity in a visual area as early as human V1 with behavior on a recognition memory test that excluded mnemonic mechanisms related to visual awareness. The results are central to broadening the scope of theoretical work on the role of early visual cortex in learning and memory, and go beyond dominant dual-process (episodic) models of recognition memory that have centered on explaining the conscious retrieval of episodic traces or familiarity mediated by the medial temporal lobe.

SUPPLEMENTAL INFORMATION

Supplemental Information includes three figures, three tables, Supplemental Experimental Procedures, Supplemental Results, and one movie and can be found with this article online at http://dx.doi.org/10.1016/j.cub.2016.01.040.

AUTHOR CONTRIBUTIONS

Conceptualization, C.R.R. and D.S.; Methodology, C.R.R.; Software, C.R.R. and D.S.; Formal Analysis, C.R.R. and D.S.; Investigation, C.R.R., S.K.A., and C.A.A.; Resources, C.R.R.; Writing – Original Draft, C.R.R.; Writing – Review & Editing, C.R.R., D.S., and C.K.; Visualization, C.R.R. and D.S.; Supervision, C.R.R. and D.S.; Project Administration, C.R.R.; Funding Acquisition, C.R.R., C.K., and D.S.

ACKNOWLEDGMENTS

All participants provided informed written consent in accordance with the terms of approval granted by the local research ethics committee and the principles expressed in the Declaration of Helsinki, and were naive to the purpose of the study. This work was supported by the Wellcome Trust (WT073735MA; C.R.R. and C.K.; http://www.wellcome.ac.uk/), the Medical Research Council (UK, 89631; D.S.; http://www.mrc.ac.uk/), the National Institute for Health Research (NIHR) Oxford Biomedical Research Centre based at Oxford University Hospitals NHS Trust and University of Oxford (C.R.R., C.A.A., and C.K.; http://oxfordbrc.nihr.ac.uk/), and the Dementias and Neurodegenerative Diseases Research Network (C.A.A.; https://www.crn.nihr.ac.uk/dementia). We are grateful for comments made by five anonymous reviewers on earlier versions of the manuscript.

Received: September 24, 2015
Revised: November 16, 2015
Accepted: January 20, 2016
Published: March 3, 2016

REFERENCES

1. Sasaki, Y., Nanez, J.E., and Watanabe, T. (2010). Advances in visual perceptual learning and plasticity. Nat. Rev. Neurosci. *11*, 53–60.
2. Eichenbaum, H., Yonelinas, A.P., and Ranganath, C. (2007). The medial temporal lobe and recognition memory. Annu. Rev. Neurosci. *30*, 123–152.
3. Clark, R.E. (2013). Recognition memory: an old idea given new life. Curr. Biol. *23*, R725–R727.
4. Albouy, G., Sterpenich, V., Balteau, E., Vandewalle, G., Desseilles, M., Dang-Vu, T., Darsaud, A., Ruby, P., Luppi, P.-H., Degueldre, C., et al. (2008). Both the hippocampus and striatum are involved in consolidation of motor sequence memory. Neuron *58*, 261–272.
5. Squire, L.R., Wixted, J.T., and Clark, R.E. (2007). Recognition memory and the medial temporal lobe: a new perspective. Nat. Rev. Neurosci. *8*, 872–883.
6. Cooke, S.F., Komorowski, R.W., Kaplan, E.S., Gavornik, J.P., and Bear, M.F. (2015). Visual recognition memory, manifested as long-term habituation, requires synaptic plasticity in V1. Nat. Neurosci. *18*, 262–271.
7. Gavornik, J.P., and Bear, M.F. (2014). Learned spatiotemporal sequence recognition and prediction in primary visual cortex. Nat. Neurosci. *17*, 732–737.
8. Voss, J.L., and Paller, K.A. (2009). An electrophysiological signature of unconscious recognition memory. Nat. Neurosci. *12*, 349–355.
9. Watkins, S., Shams, L., Josephs, O., and Rees, G. (2007). Activity in human V1 follows multisensory perception. Neuroimage *37*, 572–578.
10. Hubel, D.H. (1982). Exploration of the primary visual cortex, 1955-78. Nature *299*, 515–524.
11. Macknik, S.L., and Martinez-Conde, S. (2004). Dichoptic visual masking reveals that early binocular neurons exhibit weak interocular suppression: implications for binocular vision and visual awareness. J. Cogn. Neurosci. *16*, 1049–1059.
12. Murray, S.O., Boyaci, H., and Kersten, D. (2006). The representation of perceived angular size in human primary visual cortex. Nat. Neurosci. *9*, 429–434.
13. Smith, S.M., Jenkinson, M., Woolrich, M.W., Beckmann, C.F., Behrens, T.E., Johansen-Berg, H., Bannister, P.R., De Luca, M., Drobnjak, I., Flitney, D.E., et al. (2004). Advances in functional and structural MR image analysis and implementation as FSL. Neuroimage *23* (*Suppl 1*), S208–S219.
14. Worsley, K.J. (2001). Statistical analysis of activation images. In Functional MRI: An Introduction to Methods, P. Jezzard, P.M. Matthews, and S.M. Smith, eds. (Oxford University Press), pp. 251–270.
15. Schendan, H.E., Searl, M.M., Melrose, R.J., and Stern, C.E. (2003). An FMRI study of the role of the medial temporal lobe in implicit and explicit sequence learning. Neuron *37*, 1013–1025.
16. Chong, T.T., Husain, M., and Rosenthal, C.R. (2014). Recognizing the unconscious. Curr. Biol. *24*, R1033–R1035.
17. Rosenthal, C.R., Kennard, C., and Soto, D. (2010). Visuospatial sequence learning without seeing. PLoS ONE *5*, e11906.
18. Atas, A., Faivre, N., Timmermans, B., Cleeremans, A., and Kouider, S. (2014). Nonconscious learning from crowded sequences. Psychol. Sci. *25*, 113–119.
19. Maniscalco, B., and Lau, H. (2012). A signal detection theoretic approach for estimating metacognitive sensitivity from confidence ratings. Conscious. Cogn. *21*, 422–430.
20. Scott, R.B., Dienes, Z., Barrett, A.B., Bor, D., and Seth, A.K. (2014). Blind insight: metacognitive discrimination despite chance task performance. Psychol. Sci. *25*, 2199–2208.
21. Charles, L., Van Opstal, F., Marti, S., and Dehaene, S. (2013). Distinct brain mechanisms for conscious versus subliminal error detection. Neuroimage *73*, 80–94.

22. Jachs, B., Blanco, M.J., Grantham-Hill, S., and Soto, D. (2015). On the independence of visual awareness and metacognition: a signal detection theoretic analysis. J. Exp. Psychol. Hum. Percept. Perform. *41*, 269–276.

23. Berry, C.J., Shanks, D.R., Speekenbrink, M., and Henson, R.N. (2012). Models of recognition, repetition priming, and fluency: exploring a new framework. Psychol. Rev. *119*, 40–79.

24. Shanks, D.R., Channon, S., Wilkinson, L., and Curran, H.V. (2006). Disruption of sequential priming in organic and pharmacological amnesia: a role for the medial temporal lobes in implicit contextual learning. Neuropsychopharmacology *31*, 1768–1776.

25. Bosch, S.E., Jehee, J.F., Fernández, G., and Doeller, C.F. (2014). Reinstatement of associative memories in early visual cortex is signaled by the hippocampus. J. Neurosci. *34*, 7493–7500.

26. Turk-Browne, N.B., Scholl, B.J., Johnson, M.K., and Chun, M.M. (2010). Implicit perceptual anticipation triggered by statistical learning. J. Neurosci. *30*, 11177–11187.

27. Horton, J.C., and Hoyt, W.F. (1991). The representation of the visual field in human striate cortex. A revision of the classic Holmes map. Arch. Ophthalmol. *109*, 816–824.

28. Hinds, O.P., Rajendran, N., Polimeni, J.R., Augustinack, J.C., Wiggins, G., Wald, L.L., Diana Rosas, H., Potthast, A., Schwartz, E.L., and Fischl, B. (2008). Accurate prediction of V1 location from cortical folds in a surface coordinate system. Neuroimage *39*, 1585–1599.

29. Fischl, B. (2012). FreeSurfer. Neuroimage *62*, 774–781.

30. Benson, N.C., Butt, O.H., Datta, R., Radoeva, P.D., Brainard, D.H., and Aguirre, G.K. (2012). The retinotopic organization of striate cortex is well predicted by surface topology. Curr. Biol. *22*, 2081–2085.

31. Nissen, M.J., and Bullemer, P. (1987). Attentional requirements of learning: Evidence from performance measures. Cognit. Psychol. *19*, 1–32.

32. Censor, N., Sagi, D., and Cohen, L.G. (2012). Common mechanisms of human perceptual and motor learning. Nat. Rev. Neurosci. *13*, 658–664.

33. Cooke, S.F., and Bear, M.F. (2015). Visual recognition memory: a view from V1. Curr. Opin. Neurobiol. *35*, 57–65.

34. Serences, J.T., and Yantis, S. (2006). Selective visual attention and perceptual coherence. Trends Cogn. Sci. *10*, 38–45.

35. Chubykin, A.A., Roach, E.B., Bear, M.F., and Shuler, M.G. (2013). A cholinergic mechanism for reward timing within primary visual cortex. Neuron *77*, 723–735.

36. Karklin, Y., and Lewicki, M.S. (2009). Emergence of complex cell properties by learning to generalize in natural scenes. Nature *457*, 83–86.

37. Craik, F.I., Rose, N.S., and Gopie, N. (2015). Recognition without awareness: Encoding and retrieval factors. J. Exp. Psychol. Learn. Mem. Cogn. *41*, 1271–1281.

38. Summerfield, C., Egner, T., Greene, M., Koechlin, E., Mangels, J., and Hirsch, J. (2006). Predictive codes for forthcoming perception in the frontal cortex. Science *314*, 1311–1314.

39. Johnson, J.D., McDuff, S.G., Rugg, M.D., and Norman, K.A. (2009). Recollection, familiarity, and cortical reinstatement: a multivoxel pattern analysis. Neuron *63*, 697–708.

40. Schacter, D.L., and Buckner, R.L. (1998). Priming and the brain. Neuron *20*, 185–195.

41. Schacter, D.L., Wig, G.S., and Stevens, W.D. (2007). Reductions in cortical activity during priming. Curr. Opin. Neurobiol. *17*, 171–176.

42. Kouider, S., and Dehaene, S. (2007). Levels of processing during non-conscious perception: a critical review of visual masking. Philos. Trans. R. Soc. Lond. B Biol. Sci. *362*, 857–875.

43. López-Aranda, M.F., López-Téllez, J.F., Navarro-Lobato, I., Masmudi-Martín, M., Gutiérrez, A., and Khan, Z.U. (2009). Role of layer 6 of V2 visual cortex in object-recognition memory. Science *325*, 87–89.

44. Kirwan, C.B., Wixted, J.T., and Squire, L.R. (2008). Activity in the medial temporal lobe predicts memory strength, whereas activity in the prefrontal cortex predicts recollection. J. Neurosci. *28*, 10541–10548.

45. Viskontas, I.V., Knowlton, B.J., Steinmetz, P.N., and Fried, I. (2006). Differences in mnemonic processing by neurons in the human hippocampus and parahippocampal regions. J. Cogn. Neurosci. *18*, 1654–1662.

46. Rutishauser, U., Mamelak, A.N., and Schuman, E.M. (2006). Single-trial learning of novel stimuli by individual neurons of the human hippocampus-amygdala complex. Neuron *49*, 805–813.

47. Brown, M.W. (2008). Hippocampal and perirhinal functions in recognition memory. Nat. Rev. Neurosci. *9*, 405, author reply 405.

48. Henke, K. (2010). A model for memory systems based on processing modes rather than consciousness. Nat. Rev. Neurosci. *11*, 523–532.

Current Biology, Volume *26*

Supplemental Information

Learning and Recognition of a Non-conscious Sequence of Events in Human Primary Visual Cortex

Clive R. Rosenthal, Samantha K. Andrews, Chrystalina A. Antoniades, Christopher Kennard, and David Soto

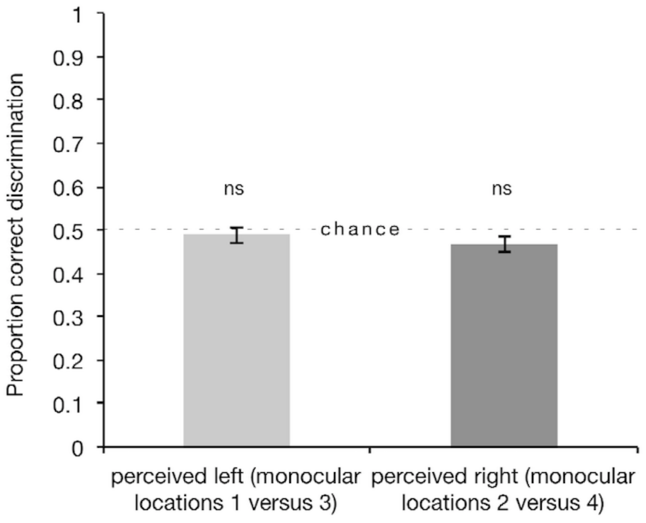

Figure S1 is related to Figure 1 (test phase, section 2c: location awareness test). Perceptual sensitivity on the location awareness test (LAT) for the four monocular locations associated with the non-conscious sequence. Participants were unable to identify the correct monocular locations of the perceived visual targets, despite extensive the perceptual training associated with repeated exposure to the non-conscious sequence during learning and recognition phases, and later instruction on the mapping between the four-monocular locations and two perceived locations, before responding on the LAT. Null sensitivity for monocular location information held when d' – a signal detection theory based measure of perceptual sensitivity – was analyzed at both perceived locations. Error bars correspond to standard error of the mean.

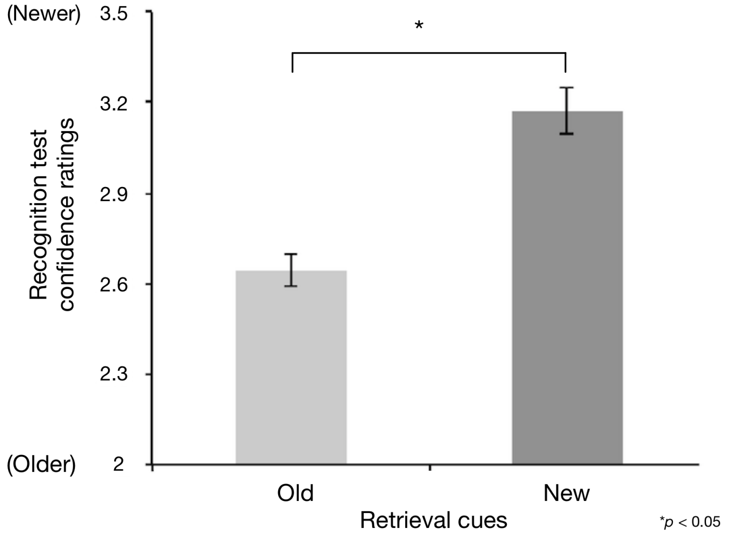

Figure S2 is related to Figure 1 (test phase, section 2b: non-conscious recognition test) and Figure 3 (behavioural results predicted by BOLD activity in V1). Recognition memory for the non-conscious sequence. Mean confidence ratings associated with the non-conscious 'old' second-order conditional and non-conscious 'new' second-order conditional

based retrieval cues were significantly different ($t_{(17)} = -7.95$, $p < 0.001$, repeated measures t-test). Error bars correspond to standard error of the mean.

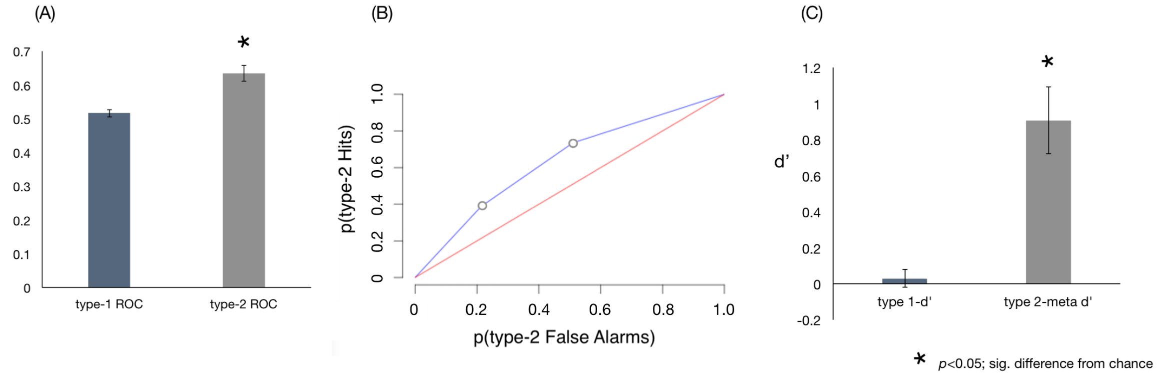

Figure S3 is related to Figure 1 (test phase, section 2b: non-conscious recognition test) and Figure 3. Type-1 and type-2 sensitivity analyses of recognition memory performance (A). Area under the ROC curve (AuC) for type-1 and type-2 performance. Type-1 sensitivity for 'old'/'new' discrimination was at chance (i.e., for sensitivity based on the area under the type-1 ROC: $t_{(17)}$=1.45, p=0.17, mean=0.52, s.e.m.=0.01, chance=0.5), whereas type-2 sensitivity was significantly above chance (for sensitivity based on the area under the type-2 ROC: $t_{(17)}$=5.79, p<0.001, mean=0.634, s.e.m.=0.02, chance=0.5) **(B)** type-2 receiver-operating-characteristic (ROC) curve (C) Analyses of d' for type-1 and type-2 performance (meta-d'). Type-1 d' was at chance ($t_{(17)}$=0.59, p=0.57, mean=0.03, s.e.m=0.05, chance=0), whereas type-2 meta-d' was significant ($t_{(17)}$=4.90, p<0.001, mean=0.9, s.e.m.=0.18, chance=0). Error bars correspond to standard error of the mean.

Supplemental Tables

Table S1 is related to Figure 1 (test phase, section 2b(ii): behavioural results from non-conscious recognition test old/new responses) and Figure 3. Proportion of 'old'/'new' responses across old (trained) and new (untrained) retrieval cues (stimuli)

	Old (trained) retrieval cue	New (untrained) retrieval cue
'Old' response	0.30	0.29
'New' response	0.20	0.21

Table S2 is related to Figure 1 (test phase, section 2b(iii): behavioural results from non-conscious recognition test confidence ratings) and Figure 3. Proportion of confidence responses across old and new retrieval cues

	Least certain	Fairly certain	Certain
Old retrieval cue	0.21	0.14	0.15
New retrieval cue	0.18	0.18	0.14

Table S3 is related to Figure 1 (test phase, section 2b(i): stimulus materials used on non-conscious recognition memory test) and Figure 3. **Masked locations of old and new retrieval cues presented on the non-conscious recognition memory test.** Old and new retrieval cues were based on two different 12-element second-order conditional sequences (SOC1 and SOC2). The old or new status of each six-item sequence derived from the respective 12-element sequences was determined by training on the dichoptic sequence learning protocol (SOC1 or SOC2). Critically, perceived binocular locations for SOC1 and SOC2 are identical across matched pairs of the six-item recognition retrieval cues, and all perceived and non-conscious structural properties were equated, along with low-level stimulus properties such as the laterality of stimuli at eye-of-origin. Masked locations 1, 2, 3, 4, read from left to right for masked four-location placeholder array. L = Left placeholder; R = Right placeholder.

Companion old and new retrieval cues used on the non-conscious recognition memory test		
Monocular locations of non-conscious cues (SOC1)	Monocular locations of non-conscious cues (SOC2)	Locations of perceived retrieval cues (i.e., perceived old and new retrieval were equated for serial order and location)
3 4 2 3 1 2	1 4 2 1 3 2	L R R L L R
4 2 3 1 2 1	4 2 1 3 2 3	R R L L R L
2 3 1 2 1 4	2 1 3 2 3 4	R L L R L R
3 1 2 1 4 3	1 3 2 3 4 1	L L R L R L
1 2 1 4 3 2	3 2 3 4 1 2	L R L R L R
2 1 4 3 2 4	2 3 4 1 2 4	R L R L R R
1 4 3 2 4 1	3 4 1 2 4 3	L R L R R L
4 3 2 4 1 3	4 1 2 4 3 1	R L R R L L
3 2 4 1 3 4	1 4 2 1 3 2	L R R L L R
2 4 1 3 4 2	2 4 3 1 4 2	R R L L R R
4 1 3 4 2 3	4 3 1 4 2 1	R L L R R L
1 3 4 2 3 1	3 1 4 2 1 3	L L R R L L

Supplemental Experimental Procedures

Participants

Eighteen undergraduate students at the University of Oxford were recruited (mean age of 21.9 years; nine female). All participants gave informed written consent to take part in accordance with the terms of approval granted by the local research ethics committee, the principles expressed in the Declaration of Helsinki, and were naive to the purpose of the

experiment. All participants reported normal or corrected-to-normal vision, no history of neurological disease, and no other contraindications for MRI.

Dichoptic presentation and visual stimuli

Presentation of the visual targets involved the use of dichoptic presentation using prism-based eyeglasses, detailed by Schurger [S1] and illustrated in Figure 1 and in Supplemental Movie 1. Participants lay supine on the scanner bed, and foam pads were used around the head to minimize head movements. Six pairs of MRI-compatible spectacles, fitted with prism lenses from 2–12 diopters in steps of 2, were available to cover disparities of 1.5, 2.3, 3.4, 4.5, 5.7, and 6.8 degree of visual angle and individual differences in fusion range. This method of dichoptic presentation was used because it eliminated crosstalk between each eye-of-origin, equated the luminance arriving at each eye, and enabled binocular fusion to be sustained for long periods [S1, S2].

The stability of binocular fusion inside the scanner was tested before each block of trials during the learning phase, the recognition test phase, the functional localiser, and on the location awareness test – the accuracy of responding served as an additional test of the efficacy of masking [S3, S4]. The test of binocular fusion involved the presentation of a short sequence of visual targets at the four fixed monocular locations (two at each eye-of-origin). Participants were required to provide an accurate online verbal report from inside the scanner of the perceived location of each of target presented at each monocular location. All participants reported only seeing two targets and reported the locations of the perceived targets accurately throughout all stages of the experiment.

All visual stimuli were presented on a computer screen (resolution: 1024 x 768 pixels), and were viewed via a mirror attached to the head coil of the MRI scanner. Each target was presented for 2000 ms at the centre of one of four monocular locations circumscribed by two horizontally aligned and isoluminant figures-of-eight (read from left to right, placeholders 1 and 2 were in the left figure-of-eight and placeholders 3 and 4 were in the right figure-of-eight). When viewed through the prism-based stereoscope, the placeholders were perceived as a single, fused, and centrally positioned figure-of-eight, aligned with the horizontal meridian. Visual targets presented at monocular locations 1 and 3 appeared within the left placeholder of the fused figure-of-eight (binocular left targets), whereas targets presented at monocular locations 2 and 4 appeared within the right placeholder (binocular right targets). Hence, on any single trial, the target stimulus was presented to the separate and independent field of view of one eye, within one of the monocular locations masked by dichoptic fusion. The eye-of-origin of visual input for each target was thereby continuously masked from visual awareness; i.e., location information for the target stimuli and thus the target visuospatial sequence were masked from visual awareness. The efficacy of visual masking was independently assessed on a location awareness test (LAT) (see below). This method of masking the monocular sequence of targets from visual awareness was continuous and did not rely on subjective report.

Behavioural tasks: Design, Materials and Procedures

The experiment was conducted in two separate main phases (Fig. 1): (1) Learning phase (dichoptic sequence learning protocol): participants were presented with a single repeating 12-element non-conscious (monocular) visuospatial sequence, based on a second-order conditional rule, and displayed at four monocular locations on a computer monitor; (2)

Test phase (a) a sequence awareness questionnaire: participants responded immediately after the learning phase to indicate whether they had detected any regularities in the sequence of stimuli; (b) dichoptic non-conscious recognition memory test: here, observers (i) viewed either an old/studied or a new/non-studied retrieval cue comprised of a sequence of six visual targets, which were presented in the same way as on the dichoptic sequence learning protocol; (ii) responded to indicate whether each retrieval cue was either old or new, with respect to the learning phase; and, (iii) rated their confidence associated with each old/new response on a six-point scale (see below). Importantly, the old and new retrieval cues differed only in terms of the masked serial order of the non-conscious sequence, and followed an identical perceived serial order (Fig. 1, Test phase; Table S2); and, (c) location awareness test (LAT): the LAT was administered to re-assess the efficacy with which location information for the target four-location visuospatial sequence was masked from visual awareness, following repeated (perceptual) exposure to the monocular sequence of stimuli during the learning and test phases (Fig. 1c).

Participants completed a practice version of the dichoptic sequence learning protocol and all four phases inside the scanner (total duration: ~2.5 hr). Participants were instructed to avoid making eye movements during learning and tests phases and during the LAT. fMRI data were acquired during phases 1 and 2b. fMRI data were also acquired during functional localiser scan for the monocular locations (see below). All of the behavioural tasks were implemented and administered using E-prime Pro (version, 2.0; Psychology Software Tools, Inc. Pittsburgh, PA).

Learning phase: dichoptic sequence learning protocol and large diameter target counting task

Participants were not informed about the repeating sequence during the learning phase, and were naive to the non-conscious monocular location of each target – separately, we have demonstrated that an intentional orientation to the sequence does not modulate learning or facilitate the detection of the non-conscious sequence [S3]. Visual targets consisted of white circles of two sizes – a standard (5 mm diameter) and a large diameter target (LDT; 8 mm diameter) – presented within one of the four monocular locations (Fig. 1) for 2000 ms.

Targets were perceived at one of two locations, and participants were asked to discriminate between the standard (5 mm diameter) and large diameter targets (LDTs; 8 mm diameter), in order to maintain a cumulative count of the number of LDTs on each block and perform within 5% accuracy [S3, S5]. Presentation of LDTs occurred on 17-35% of trials on both sequence and pseudorandom baseline blocks (see below) – the order of the LDTs was random within a block and set at a proportion to ensure ceiling level performance. The LDT counting task was performed concurrently with the dichoptic sequence learning protocol. On-screen feedback - in the form of percentage under- or over-estimation - at the end of each block of trials was provided immediately after entering a value on the LDT counting task.

Notably, performance on the LDT counting task during the learning phase provided an additional and independent source of data regarding the stability of binocular fusion inside the scanner, because ceiling performance was dependent on stable fusion [S3, S4]. An initial practice block comprised of 30 trials was administered inside the scanner to ensure task comprehension and accurate performance on the LDT counting task. During the learning,

blocks were separated by a 30 s response period to allow a value on the LDT counting task to be entered by the participant.

Each sequence block was comprised of eight repetitions of the same 12-element second-order conditional sequence (SOC) specified at the four monocular locations. Two SOCs were counterbalanced across participants to control for sequence-specific effects (SOC1: 3-4-2-3-1-2-1-4-3-2-4-1; SOC2: 3-4-1-2-4 3-1-4-2-1-3-2; positions 1-4 are read from left to right of the masked four-location array, with the spatial locations corresponding to numeric values). Half of the observers studied SOC1 and the other half studied SOC2. Each SOC sequence was equated along dimensions that ensured learning was directed towards the SOC rule; namely, simple frequency of location, laterality, frequency of first-order transition, reversal frequency (e.g., 1-2-1), and rate of full coverage. The two SOC sequences differed only at the level of serial order of three or more consecutive positions within the sequence, where, at the lowest structural level, the ability to predict a target position is dependent on learning two preceding monocular target positions. Unlike a first-order sequence, performance cannot improve from learning the frequencies of individual positions or pairs of positions. Binocular perception of the visual targets during the learning phase was consistent with the following sequences of left(L)/right(R) perceived locations: SOC1: L-R-R-L-L-R-L-R-L-R-R-L; and, SOC2: L-R-L-R-R-L-L-R-R-L-L-R.

The learning phase was implemented in three runs of fMRI acquisition, separated by breaks of approximately 5 min (Fig. 1). Runs 1 and 2 were comprised of four sequence blocks (S; 100 trials/block) and two baseline blocks (R; 50 trials/block) (SSRSRS). Run 3 was comprised of five sequence blocks and two baseline blocks (SSRSRSS). An additional sequence block was included at the end of the run to facilitate learning of the target non-conscious sequence.

The structure of the blocks during the learning phase was designed to facilitate rapid acquisition of sequence-specific SOC knowledge. The serial order of the visual targets on the baseline (unstructured) blocks was pseudorandom, and was constrained so that the same monocular location did not appear consecutively and targets appeared with equal frequency at each of the four monocular locations. Therefore, the serial order of structured and pseudorandom blocks were visibly different (i.e., at the perceived two-location L-R locations). We elected to use a pseudorandom sequence, instead of a non-trained second-order conditional sequence on baseline blocks to facilitate learning of the target SOC sequence blocks.

Sequence awareness questionnaire

Awareness associated with learning phase was tested on a post-learning phase awareness questionnaire administered during the break between learning and test scanning phases (see below). Each participant was instructed to select one of the following five propositions: 1 = "The sequence of stimuli was random"; 2 = "Some positions occurred more often than others; 3 = "The movement of the stimuli was often predictable"; 4 = "The same sequence of stimuli would often appear"; and 5 = "The same sequence of stimuli occurred throughout the experiment" [S6].

Dichoptic non-conscious recognition memory test

The dichoptic recognition memory test was administered approximately 15-20 minutes after completion of the dichoptic sequence learning protocol. Each trial on the recognition test was comprised of three stages. **(i)** Participants were shown either an old or a new retrieval cue (six-element sequences; 12 old and 12 new) in a random order (7.2 s; Fig. 1b). Stimuli that comprised the retrieval cue were presented and viewed in the same way as on the dichoptic sequence learning protocol. **(ii)** One second after the offset of each retrieval cue, a prompt appeared for the participants to identify the sequence as either 'old' or 'new' (8 s) (with respect to the learning phase). It was emphasized to participants that a proportion of the sequences had been studied, and an 'old' response should be given if they felt any sense that the retrieval cues were at all familiar, using whatever minimal information was available to aid accurate identification of the old sequences. **(iii)** Participants then provided a value on a confidence rating scale to indicate the confidence in their response (8 s) [S7]: for retrieval cues designated as, old: 1 = "I'm certain that this fragment was part of the training sequence"; 2 = "I'm fairly certain that this fragment was part of the training sequence"; 3 = "I believe that this fragment was part of the training sequence"; for retrieval cues designated as new: 4 = "I believe that this fragment was not part of the training sequence"; 5 = "I'm fairly certain that this fragment was not part of the training sequence"; 6 = "I'm certain that this fragment was not part of the training sequence." The scale was designed to maximise response distance between old and new retrieval cues, and encourage participants to consider the scale in full.

Twelve six-element retrieval cues (starting from each ordinal position of the 12-element SOC sequence for six consecutive locations) were generated from SOC1 and 12 six-element recognition sequences were generated from SOC2 (see Table S3). These were assigned as old and new retrieval cues in accordance with the counterbalancing described earlier. For each old retrieval cue, the first predictable target occurred after 2.4 s (i.e., the onset of the third target in each six-element sequence); correspondingly, the third stimulus provided the first potential source of discrimination between the old and new retrieval cues (i.e., on the basis that the non-conscious second-order conditional subsequences were all unique to the trained SOC).

The non-conscious recognition memory test was implemented as a slow event-related design (24.2 s for each trial), with variable inter-item lags between each trial to permit efficient segregation of the hemodynamic responses. Manual key presses on one of four keys mapped to the right hand were used to indicate the number of LDT targets presented on the immediately preceding block of trials, during the 30 s count period between blocks of trials. The mapping was implemented to minimise and equate key presses, as far as possible, across each response period. Manual responses were recorded using a fibre optic Lumina Response Pad (LU444-RH, Cedrus Corporation, San Pedro, USA) connected to a PC running E-Prime Pro.

There are two remarkable features of this recognition test. First, the monocular spatial information necessary to distinguish old retrieval cues (e.g., 3-4-1-2-4-3) from new retrieval cues (e.g., 1-4-3-2-4-1) was not available to visual awareness (as confirmed by signal detection theory based analyses of performance conducted on the results from the location awareness test, which was administered after the recognition test). Second, the old and new retrieval cues were both perceived in the same binocular serial order. In particular, for the two example sequences, old and new retrieval cues were perceived as the same, L-R-L-R-R-L, binocular sequence, and so differed only in terms of the masked serial order of the respective old and new second-order conditional sequences (Table S3).

An additional important consequence of matching the structural properties of the old and new retrieval cues – i.e., simple frequency of location, laterality, first-order transition frequency, reversal frequency, presence of second-order conditional serial dependencies and rate of full coverage of the four locations – is that it provided an effective basis on which to study neural and behavioural effects related to recognition under conditions that did not reflect 'old'/'new' differences in the spatial allocation of attention or low-level old-new differences, such as laterality at the eye-of-origin. As an example of the latter, for old and new retrieval cues perceived as, L-R-L-R-R-L, the underlying non-conscious old sequence – 3-4-1-2-4-3 – and new sequence – 1-4-3-2-4-1 – were equated in terms of laterality from each eye-of-origin (3 positions from the left eye-of-origin; 3 positions from the right eye-of-origin).

To summarise, this setup allowed us to base the non-conscious recognition test on a contrast between the studied non-conscious sequence and an non-studied/untrained non-conscious (second-order conditional) sequence that was equated in terms of the perceived serial order and all other structural dimensions of the sequence, apart from the four-location monocular serial order of the non-conscious second-order conditional retrieval cues. The number and six-element format of retrieval cues corresponds with established studies of sequence learning based on the use of a visible 12-element SOC [e.g., S8]. Hence, the recognition memory test assessed observers' sensitivity to the non-conscious second-order conditional properties acquired during the dichoptic sequence learning phase.

Location Awareness Test (LAT)

Visual targets were viewed through the prism-based stereoscopic setup inside the scanner, in the same way as during the dichoptic sequence learning and recognition test phases. The mapping between monocular targets and their appearance at the two perceived locations was explained to the participants in order to encourage accurate trial-by-trial forced-choice responses, and improve sensitivity to even partial knowledge of the location of the monocular stimuli, because this could serve as a basis for accurate detection of the location of each monocular stimulus. Forced-choice responses were performed for targets that appeared within the left binocular location (i.e., choose between masked locations 1 and 3) and for targets that appeared at the right binocular location (i.e., choose between masked locations 2 and 4). Targets remained on screen until a manual response was entered on the response pad, and were separated by a 200 ms ISI. Two blocks of forced-choice probe trials were used to test perceptual sensitivity at all four monocular locations on each block; all four monocular locations were represented equally on the LAT.

An inability to identify masked locations provided evidence that the locations of stimuli presented at the four monocular locations – and thus information necessary to consciously learn the SOC associations specific across eye-of-origin – were masked from visual awareness. More generally, for a 12-element SOC, such location information would need to be continuously available at each of the four monocular locations in order for the sequence to be learned in the same way as on a standard serial reaction time task, which is typically used to investigate conventional somatomotor "implicit" sequence learning at four visible locations [e.g., S9, S10, S11].

Functional MRI localizer for monocular locations

All participants also underwent an fMRI-based localiser scan to identify voxels in the early visual cortex associated with stimulus input at the monocular locations, used to present the sequences on the separate learning and recognition tests. Participants were informed that four blocks of trials (50 trials/block, randomised) would be presented, and targets would appear at a single monocular location during each block (200 ms ISI). Participants were encouraged to keep their eyes fixated at the center of the placeholder, and their heads were fixed in position with bilateral bracing with soft foam pads, as in all other stages of the experiment. Participants were also instructed to perform the LDT counting task and respond with a value in the same way as during the response period after each block of stimuli. The LDT counting task was used to ensure that participants attended to the stimuli at each single monocular location. Blocks were separated by a 30 s response period to allow a cumulative value on the LDT counting task to be entered.

Behavioural Eye Tracking Control Experiment: Eye movements during dichoptic sequence learning

To assess the role of voluntary (which countermanded the instruction to fixate) and involuntary eye movements, we conducted a separate behavioural experiment in which eye movements were recorded from both eyes using an infrared eye tracker, at a sampling frequency of 1kHZ and 12 bits resolution (Jazz Novo multisensor eye tracker, Ober Consulting; spatial resolution: 0.1°). Participants were asked to maintain central fixation in order to discourage voluntary eye movements. The experimental design was the same as used in the dichoptic fMRI experiment. The eye tracker was calibrated to each participant at the start of the learning and test phases. Visual fixation was assessed by calculating the frequency of eye movements (saccades) outside of central fixation (>1°), using a minimum velocity criterion of 30 degrees per second. Analysis was carried out using Jazz Manager (Ober Consulting).

Direction of saccades and relationship to the two-location perceived sequence and monocular four-location non-conscious sequence: Eye Movement Analyses

The criteria used to mark the onset and offset of conjugate saccades are similar to those used by other studies [S12]. The onset of the saccade was defined as the time when the eye velocity exceeded 5% of the peak saccadic velocity and the offset was defined as the point at which eye velocity dropped below 100/s. Eye movements with latencies longer than 1000 ms along with express eye movements with very short latencies (< 80 ms) were also rejected.

fMRI control experiment: Experimental procedures

Participants

Seventeen undergraduate students were recruited (mean age of 23±2 years; nine female). All participants gave informed written consent to take part in accordance with the terms of approval granted by the local research ethics committee, the principles expressed in the Declaration of Helsinki, and were naive to the purpose of the study. All participants reported normal or corrected-to-normal vision, no history of neurological disease, and no other contraindications for MRI.

Methods

In the control fMRI study, four fixed sequence locations were available to visual awareness and were presented using the same MRI-compatible display system as described below for the main study; hence, dichoptic masking was not used. The experiment was designed to test for commonalities and differences in the learning-related effect, as measured by the high-level contrast between structured and pseudorandom blocks, described in the main text and below, in relation to the analyses of the learning phase. In line with the dichoptic learning protocol, there were three training runs, with an identical arrangement of structured and pseudorandom sequences (see below). The only difference was that learning runs comprised 356 volumes, and each structured and pseudorandom block lasted for 60 seconds. In keeping with to the dichoptic learning protocol, participants were exposed to a repeating 12-element second-order conditional visuospatial sequence displayed at four visible locations (SOC sequences were counterbalanced, and based on the same serial order as described below in the dichoptic learning protocol). Participants were instructed to count the number of trials with a large diameter target in order to ensure that attention was sustained to the visible sequence of stimuli. All parameters of the LDT task were the same as on the dichoptic sequence learning protocol. Observers were also directed to maintain central fixation, and eye movements were monitored during scanning to ensure adherence to the instruction to maintain fixation (XY MRI Oculomotor, Ober Consulting). Infrared reflectance was digitized at 12-bit resolution. Acquisition rate was 250Hz. Minimum resolution in both vertical and horizontal was 0.1 degree. Linearity was assured for ±15°. The eye tracker was calibrated before each run at fixation (central position) and 8 eccentric points. Analyses of eye position showed that participants did not saccade to the target locations of the visible sequence (mean = 2.3, saccades/block of trials). Hence, the learning protocols with and without dichoptic masking were equated in terms excluding eye movements that coincided with the structure of the SOC visuospatial sequence, and manual responses were not performed in relation visuospatial sequence.

Functional and structural MRI data acquisition

Functional MRI series were acquired using a 3.0-Tesla scanner fitted with a 32-channel head coil (Siemens, Verio, Erlangen, Germany) in the main study and fMRI control experiment. The first two runs of the learning phase consisted of 504 volumes, whereas run 3 consisted of 600 volumes due to the additional sequence block. Three hundred volumes were acquired on the recognition test and 250 volumes were acquired on the functional localiser.

Functional volumes consisted of multi-slice T2*-weighted echoplanar images (EPI) with blood oxygenation level dependent (BOLD) contrast. We used the following scanning parameters to achieve whole brain coverage: TR = 2500 ms, TE = 30 ms, 39 coronal slices, 3 mm slice thickness, 0% interslice gap, and FoV = 220 x 220 mm. The resulting voxel size was 3.0 x 3.0 x 3.0 mm. To facilitate anatomical localization and cross-participant alignment, a high-resolution whole-brain structural T1-weighted three-dimensional magnetization-prepared rapid gradient echo (MP-RAGE) scan was acquired for each participant after the EPI imaging (176 sagittal slices TR = 1900 ms, TE = 2.48 ms, FoV= 250 x 250 mm, voxel size = 1.0 x 1.0 x 1.0 mm).

fMRI Image Preprocessing

Image preprocessing and statistical analysis of whole-brain fMRI data were carried out using FEAT (FMRI Expert Analysis Tool; version 5.98), part of the FSL (FMRIB

software library, version 5) [S13]. The Brain Extraction Tool (BET) was used to segment brain matter from non-brain [S14]. The first 6 volumes of each run of the behavioural tasks were discarded to allow for magnetic saturation effects/MR signal stabilization. Image pre-processing involved realignment of EPI images to remove the effects of movement between scans using MCFLIRT [S15], spatial smoothing using a 6-mm full-width-half-maximum Gaussian kernel, pre-whitening using FILM and high-pass filter with a cut-off frequency of 1/100 Hz. Translational movement parameters did not exceed 1 voxel in any direction for any participant or session. Images were registered to the high-resolution structural image (7 degrees of freedom) and then the standard MNI152 template, using affine registration (12 degrees of freedom) [S15]. Statistical analyses were performed in the native image space, with the statistical maps normalized to the standard space prior to higher-level analysis.

fMRI Statistical Analyses

Dichoptic sequence learning phase

The learning data were analyzed using voxel-wise time series analysis within the framework of the General Linear Model (GLM). First-level general linear model analyses for each individual run were performed with the following explanatory variables (EVs) along with their temporal derivatives: sequence (structured) blocks and baseline (unstructured, pseudorandom) blocks, using the FILM module of FSL with local autocorrelation correction [S16]. The design matrix was generated with a synthetic hemodynamic response function modeled as a double gamma function.

Sequence blocks were modeled in chunks of 48 trials (i.e., S_A and S_B) for the purposes of contrast with pseudorandom baseline blocks (48 trials); the value was selected as a multiple of 12-element unit of SOC sequence that allowed the number of trials on sequence blocks to be equated with the baseline blocks. A 30 s interval associated with the response period on the LDT counting task followed each sequence and baseline block. We performed within-subject cross run (fixed-effects) analyses testing for two separate linear contrasts (indicated by the bold text and underlined letter of the 48 trial chunk below) performed on the first-level parameter estimates: one based on corresponding chunks of sequence blocks across the three runs of the learning phase (i.e., run 1 [S_{AB} S_{AB} R S_{AB} R $\underline{\mathbf{S}}_{\underline{\mathbf{A}}B}$] < run 2 [$S_{AB}$ S_{AB} R S_{AB} R $\underline{\mathbf{S}}_{\underline{\mathbf{A}}B}$] < run 3 [S_{AB} S_{AB} R S_{AB} R $\underline{\mathbf{S}}_{\underline{\mathbf{A}}B}$ S_{AB}]); the other examined the reciprocal contrast (i.e., 1 0 -1). Each EV specified the onset and duration of each chunk block. The output of these linear contrasts across the three learning runs (e.g., -1 0 1) was fed into a mixed-effects model for whole-brain group analysis, using FLAME (Local Analysis of Mixed Effect) stage 1 and 2 [S17, S18], testing for consistent learning effects across participants. Group Z (Gaussianized T) statistic images were thresholded using clusters determined by $Z > 2.3$ and a corrected cluster extent significance threshold of $p = 0.05$, using Gaussian Random Field Theory.

It is important to note that because the pseudorandom baseline sequences were visibly different (i.e., at the perceived conscious level) from the repeating structured (second-order conditional) non-conscious sequence, the contrast of BOLD responses between structured and blocks pseudorandom during learning could reflect recognition of the difference in the perceived serial order, rather than sensitivity to the structured non-conscious sequence, per se. Therefore, in order to directly compare the learning effect of the structured sequence relative to the pseudorandom baseline, we used the following mass-univariate approach.

We computed *within-run* statistical maps that were expected to be sensitive to the nature of structured and pseudorandom sequences. Hence, we derived brain maps for the repetition of the structured sequence (i.e. S3>S4: [S1-S2-R1-**S3**-R2-**S4**]) on each of the three training runs. Correspondingly, we derived separate brain maps for the repetition of the pseudorandom sequence during a run (i.e. R1>R2: [S1-S2-**R1**-S3-**R2**-S4]) on each of the three training runs. The respective within-run estimates for the structured and for the pseudorandom sequences were submitted separately to across-run within-subjects fixed effects analyses, testing for linear modulations across the three training runs. Finally, we performed a group-level paired t-test to assess which brain regions were associated with increased training effects in the structured relative to the pseudorandom sequence (and vice versa).

Notably, unlike the recognition test, attention could have acted as an enabling condition during the learning phase, because participants could have allocated attentional resources to the perceived L-R positions in a different manner when encountering the pseudorandom blocks interspersed amongst the repeating non-conscious structured sequence blocks. Therefore, this analysis allows for the direct comparison of the learning effects for structured and pseudorandom sequences, while addressing the confound derived from fact that the sequence types were perceptually different, because structured and pseudorandom sequences were not directly compared at the lower-level of analysis (i.e., within a run). Additional analyses to address this issue are presented in the main text.

Dichoptic non-conscious recognition memory test

The onsets of the old and new retrieval cues were modeled as explanatory variables in the first-level analysis to estimate within-subject differences in BOLD responses. The duration of these EVs corresponded to the duration of each stimulus sequence (retrieval cues) plus the 8 s period assigned for an 'old'/'new' trial. Given that the retrieval cues had a long duration (7.2 seconds), alongside the need to provide exposure to at least two stimuli to enable the first SOC triplet to be recognised, with recognition of additional SOC triplets triggered by further individual stimuli [S8], and masking of the salient information from visual awareness, we elected to include the old/new response period in the model because participants' reflective processes during the old/new decision period may be relevant here to guide non-conscious recognition memory.

Note that two regressors of no interest were also included in the design matrix to control for variation in decision response time for the old/new response period and response period associated with the confidence rating. Two contrasts of parameter estimates were derived from the first-level analyses (old < new sequence: -1 1; old > new 1 -1), which were subsequently submitted to a higher-level mixed effects model testing for consistent effects across participants.

The mass-univariate analyses described above were also implemented on an individual basis using V1 region-of-interest. Individual V1 masks were derived by combining cortical activity maps in a functional localiser of the sequence locations (see below), with an automatic estimate of the location of V1 from cortical folds that was obtained using Freesurfer (http://surfer.nmr.mgh.harvard.edu/), following the method developed by Hinds and colleagues [S19].

Functional MRI localizer for monocular locations

Each of the four monocular locations was modeled as separate EVs in the first-level of the design matrix, along with the response period on the LDT counting task. We used statistical contrasts for the difference in activity between the left and right monocular locations and derived statistical maps of voxels for each individual. Finally, an individual V1 mask was derived from the intersection of voxels that exhibited activity on the functional localiser and the V1 space delineated by Freesurfer.

Analyses of functional connectivity associated with non-conscious recognition memory

In order to assess whether functional connectivity between the hippocampus and V1 was modulated during the recognition test, we used a seed-voxel based approach based on psychophysiological interactions (PPI) to identify signals of interest that would be missed in standard subtraction based analyses. A mask of the hippocampus was drawn from the responsive voxels in the old<new mass-univariate contrast, and this mask was used to define the seed region's time course for the PPI. We then estimated a model for each participant that included psychological EVs for the onsets of old and new trials, EVs for the recognition and confidence periods (as described above for the mass-univariate analysis), and, critically, for the PPI model, a physiological regressor for the time course of the region-of-interest, and psychological x physiological interaction EVs for the PPI. These new regressors were, therefore, added to the previous first-level model for each participant/run. Parameter estimates for old vs. new trials based on the PPI regressors were derived in a similar fashion to the analyses outlined above (e.g., using both fixed-effects analysis across runs followed by mixed-effect analysis across participants), which, here, directly compared the changes in functional coupling associated with training. Given our *a priori* interests in primary visual cortex, this analysis used a region-of-interest approach, with a target occipital mask.

Supplemental Results

Results of the LDT counting task

Mean accuracy in the LDT counting task during the sequence learning phase was 98.2% (S.E.M.= 0.83), and performance did not vary as a function of block type (sequence vs. baseline) ($F_{(1,17)} < 0.1$), or, across the learning phase ($F_{(18,306)} = 1.23, p = 0.24$). These data provide evidence of the ability of participants to sustain attention to the onset of targets at the two perceived locations.

Results of post-learning phase awareness questionnaire

Immediately after the dichoptic learning phase, we administered a debriefing questionnaire. Participants exhibited little or no knowledge about the sequence (M rating on the awareness questionnaire = 1.5 [the sequence was "random"], S.E.M.=0.27), and the knowledge expressed was not significantly different from random (=1) ($t_{(17)} = 1.84, p>0.05$). These data are in agreement with the results from the LAT (see above, Section 1.1), and demonstrate that *ex post facto* knowledge about even the overall basic structure of the sequence was below subjective threshold.

Results of the signal-detection analyses of the non-conscious (dichoptic) recognition memory test

A receiver-operating-characteristic (ROC) based analysis [S20, S21] was applied to measure type-1 and type-2 sensitivity. In order to conduct the type-1 perceptual sensitivity analyses (i.e., ability to distinguish stimuli), the 'signal' and 'noise' were defined as old and new retrieval cues, respectively. A 'hit' was, therefore, a correct response ('old') to an old trained (six-element sequence) retrieval cue and a 'correct rejection' was a correct response ('new') to a new untrained (six-element sequence) retrieval cue. A 'false alarm' was an incorrect response ('old') to a new retrieval cue and a 'miss' was an incorrect response ('new') to an old retrieval. To obtain the type-1 ROC curves, we plotted the probability of hits as a function of the probability of false alarms for all possible decision criteria. The different points in the ROC curve were obtained by calculating cumulative probabilities for hits and false alarms along a recoded confidence continuum ranging from 3 (i.e., *certain*) to 1 (i.e.. *least certain*) in signal present trials (old retrieval cues), and from 3 (i.e., *certain*) to 1 (i.e., *least certain*) in noise trials (new retrieval cues). On the basis of simple geometry, we computed the area under the ROC curve as a distribution-free measure of the discriminability [S22].

In order to conduct the type-2 meta-d' sensitivity analyses, the area under the ROC curve was calculated by plotting the cumulative probability of correct responses (either hit or correct rejection) and the cumulative probability of incorrect responses (either false alarm or miss) across the different levels of confidence. The area under the ROC curve was estimated as distribution-free measure of metacognitive ability [S23]. Type-1 d' and meta-d' were computed [S24]. Meta-d' is a parametric estimation of type-2 sensitivity predicted from the type-1 signal detection theoretic (SDT) model, computed by fitting a type-1 SDT model to the observed type-2 performance data and estimating the type-2 ROC curves. It corresponds to the efficacy with which the observers' confidence ratings discriminated between their own correct and incorrect stimulus classifications. All data processing and analyses were completed using R (version 3.2.2) [S25] and Matlab (The MathWorks Inc.).

The results are depicted in Figure S3, which includes plots of the area under the type-1 and type-2 curves, including an illustration of the type-2 ROC and also type-1 d' and type-2 meta d' (see below). Table S1 (see above) shows the proportion of 'old'/'new' responses for old(trained) and new(untrained) retrieval cues. Table S2 (see above) also shows the proportion of responses for each confidence rating.

Results of the location awareness test (LAT).

All participants who took part in the fMRI study were administered the LAT on completion of the dichoptic non-conscious recognition memory test. Performance on the LAT was first assessed by calculating a measure of perceptual sensitivity, d' [S26]. Each response was scored as follows: one of the responses (e.g., '1') was treated as signal present and the other response (e.g., '3') as signal absent. Thus, responding with '1' to targets at position 1 was labeled as a, hit, whereas responding with 1 to targets at position 3 was recorded as a, false alarm. The same procedure was applied in the case of 'right' side perceived targets at positions 2 and 4. In this way, we obtained the probability of hits – P(H) – and false alarms – P(FA) – to calculate, d'.

There was no evidence of conscious access to the information that could support accurate identification of the monocular locations, when the proportions of correct responses were analyzed. In particular, participants were at chance (0.5) when identifying the locations of monocular targets that appeared at locations 1 and 3 (i.e., location information in perceived

left targets; Mean (M) proportion correct responses = 0.48, S.E.M. = 0.02; $t_{(17)} = -0.63$, $p = 0.53$) and at locations 2 and 4 (i.e., location information in perceived right targets; M = 0.47, S.E.M. = 0.02, $t_{(17)} = -1.49$, $p = 0.15$). These results are depicted in Figure S1. Repeated measures t-tests indicated that sensitivity scores for perceived left targets ($t_{(17)} = -0.41$, $p = 0.68$) and for perceived right targets ($t_{(17)} = 0.58$, $p = 0.57$) did not differ from chance (d' = 0), despite extensive perceptual training and information about the mapping between the four-monocular locations and two perceived locations, before responding on the LAT.

In keeping with standard criteria for the absence of visual awareness, namely d'=0 [S27], the null sensitivity observed in the LAT indicates that observers could not identify the monocular locations that specified the SOC sequence. This was predicted because our experimental protocol provided continuous masking of the monocular sequence locations through binocular fusion. Moreover, these data also broadly align with ceiling performance on the LDT counting task, which provides an independent source of data regarding the stability of binocular fusion inside the scanner, because ceiling performance is dependent on stable fusion [S3, S4]. Finally, the results from the LAT also align with phenomenological reports from the participants who consistently reported in the post-test debriefing and dichoptic calibration sessions that two target locations were experienced in their visual field.

In a revised version of the LAT, inclusion of a confidence rating after each response could be used to assess whether such ratings align with the null sensitivity found on proportion correct and d'. Importantly, if confidence ratings or type-2 sensitivity were diagnostic of item location in the presence of null d', this would arguably be consistent with a process that is dissociable from visual awareness [S28, S29], given that d' was null when probing location information on the LAT. Such a pattern would indicate that non-conscious coding of location information, as well as non-conscious coding of more complex (SOC) associations, are both capable of yielding insight into higher-order non-conscious recognition processes.

In summary, these results suggest that our dichoptic experimental protocol was effective at masking the location information associated with the non-conscious sequence from visual awareness, and, are consistent with our previous behavioural studies [S3, S4].

Results of the eye tracking control experiment

A separate control behavioural experiment was conducted to test whether eye movements were associated with the non-conscious sequence. In the same way as in the main fMRI experiment, participants were instructed to maintain central fixation, and did not perform manual responses during the dichoptic learning and test phases. We examined whether the structure of voluntary and involuntary motor responses (eye movements that violate the instruction to fixate) were correlated with the non-conscious visuospatial sequence specified at the four masked monocular locations or with the visible two-location sequence appearing at the two perceived locations. This question was highly relevant to understanding whether dichoptic sequence learning protocol did, in fact, operate in the absence motor-based learning mechanisms. Notably, the results from the eye movement data cannot exclude a role for responses based on covert reorienting of visuospatial attention, because it remains conceivable that the structure of covert reorienting of visuospatial attention coincided with the perceived or masked visuospatial sequence. Future studies based on analyses of the structure of microsaccades during dichoptic presentation may provide further insight into this issue [S4, S30].

The results demonstrated that participants' maintained visual fixation. Even when involuntary eye movements occurred (despite the instruction to fixate), their direction, and, thus structure, was uncorrelated with the structure of the perceived two-location sequence or with the non-conscious sequence presented at the four monocular locations. The frequency of saccades (i.e., eye movements that violated the instruction to maintain central fixation) during the learning phase did not differ as a function of run or block type (repeated measures ANOVA, p=0.9, and, p=0.89, respectively), and there was no significant interaction between run and block (F<1). These results rule out the possibility that the reported learning effects were caused by differences in eye movements between sequence and baseline blocks. Furthermore, neither the direction of saccades and appearance of stimuli at the four monocular locations, nor, the appearance at the two perceived locations were significant correlated (monocular: r=0.13, p>0.05; perceived: r=0.05, p>0.05). Therefore, the results from this proxy for the allocation of attention, based on the recording eye movements during dichoptic presentation [S31], suggest that sequence learning in our paradigm operated without motor learning based mechanisms, because motor responses were uncorrelated with the structure of the four-location non-conscious or two-location perceived visuospatial sequence.

Supplemental References

S1. Schurger, A. (2009). A very inexpensive MRI-compatible method for dichoptic visual stimulation. J Neuro Methods *177*, 199-202.

S2. Schurger, A., Pereira, F., Treisman, A., and Cohen, J.D. (2010). Reproducibility distinguishes conscious from nonconscious neural representations. Science *327*, 97-99.

S3. Rosenthal, C.R., Kennard, C., and Soto, D. (2010). Visuospatial sequence learning without seeing. PloS One *5*, e11906.

S4. Rosenthal, C.R., Antoniades, C.A., Kennard, C., and Soto, D. (2013). Saccades do not reflect the learning and recognition of an unseen sequence of events. Neuro-Ophthalmol. *37(S1)*, 1–109.

S5. Rosenthal, C.R., Roche-Kelly, E.E., Husain, M., and Kennard, C. (2009). Response-dependent contributions of human primary motor cortex and angular gyrus to manual and perceptual sequence learning. J Neurosci *29*, 15115-15125.

S6. Curran, T. (1997). Effects of aging on implicit sequence learning: Accounting for sequence structure and explicit knowledge. Psychol Res *60*, 24-41.

S7. Shanks, D.R., and Johnstone, T. (1999). Evaluating the relationship between explicit and implicit knowledge in a sequential reaction time task. J Exp Psychol Learn Mem Cogn *25*, 1435-1451.

S8. Shanks, D.R., Channon, S., Wilkinson, L., and Curran, H.V. (2006). Disruption of sequential priming in organic and pharmacological amnesia: a role for the medial temporal lobes in implicit contextual learning. Neuropsychopharmacol *31*, 1768-1776.

S9. Nissen, M.J., and Bullemer, P. (1987). Attentional requirements of learning: evidence from performance measures. Cogn Psychol *19*, 1-32.

S10. Schendan, H.E., Searl, M.M., Melrose, R.J., and Stern, C.E. (2003). An fMRI study of the role of the medial temporal lobe in implicit and explicit sequence learning. Neuron *37*, 1013-1025.

S11. Albouy, G., Stephenich, V., Balteau, E., Vandwalle, G., Deseilles, M., Dang-Vu, T., Darsaud, A., Ruby, P., Luppi, P.-H., Degueldre, C., et al. (2008). Both the hippocampus and striatum are involved in consolidation of motor sequence memory. Neuron *58*, 261-272.

S12. Takagi, M., Frohman, E.M., and Zee, D.S. (1995). Gap-overlap effects on latencies of saccades, vergence and combined vergence-saccades in humans. Vision Res *35*, 3373-3388.

S13. Smith, S.M., Jenkinson, M., Woolrich, M.W., Beckmann, C.F., Behrens, T.E., Johansen-Berg, H., Bannister, P.R., De Luca, M., Drobnjak, I., Flitney, D.E., et al. (2004). Advances in functional and structural MR image analysis and implementation as FSL. Neuroimage *23 Suppl 1*, S208-219.

S14. Smith, S.M. (2002). Fast robust automated brain extraction. Hum Brain Mapp *17*, 143-155.

S15. Jenkinson, M., and Smith, S. (2001). A global optimisation method for robust affine registration of brain images. Med Image Anal *5*, 143-156.

S16. Woolrich, M.W., Ripley, B.D., Brady, J.M., and Smith, S.M. (2001). Temporal autocorrelation in univariate linear modelling of fMRI Data. NeuroImage *14*, 1370-1386.

S17. Woolrich, M.W., Behrens, T.E., Beckmann, C.F., Jenkinson, M., and Smith, S.M. (2004). Multilevel linear modelling for FMRI group analysis using Bayesian inference. Neuroimage *21*, 1732-1747.

S18. Beckmann, C.F., Jenkinson, M., and Smith, S.M. (2003). General multilevel linear modeling for group analysis in FMRI. Neuroimage *20*, 1052-1063.

S19. Hinds, O.P., Rajendran, N., Polimeni, J.R., Augustinack, J.C., Wiggins, G., Wald, L.L., Diana Rosas, H., Potthast, A., Schwartz, E.L., and Fischl, B. (2008). Accurate prediction of V1 location from cortical folds in a surface coordinate system. Neuroimage *39*, 1585-1599.

S20. Egan, J.P. (1975). Signal detection theory and ROC-analysis, (New York: Academic Press).

S21. Green, D.M., and Swets, J.A. (1966). Signal detection theory, (Huntington, New York: Wiley).

S22. Kornbrot, D.E. (2006). Signal detection theory, the approach of choice: model-based and distribution-free measures and evaluation. Percept Psychophys *68*, 393-414.

S23. Fleming, S.M., Weil, R.S., Nagy, Z., Dolan, R.J., and Rees, G. (2010). Relating introspective accuracy to individual differences in brain structure. Science *329*, 1541-1543.

S24. Maniscalco, B., and Lau, H. (2012). A signal detection theoretic approach for estimating metacognitive sensitivity from confidence ratings. Conscious Cogn *21*, 422-430.

S25. Team, R.D.C. (2008). R: A language and environment for statistical computing. (Vienna, Austria: R Foundation for Statistical Computing).

S26. Wickens, C. (1992). Engineering psychology and human performance, (New York: Harper Collins).

S27. Dehaene, S., and Changeux, J.P. (2011). Experimental and theoretical approaches to conscious processing. Neuron *70*, 200-227.

S28. Charles, L., Van Opstal, F., Marti, S., and Dehaene, S. (2013). Distinct brain mechanisms for conscious versus subliminal error detection. Neuroimage *73*, 80-94.

S29. Jachs, B., Blanco, M.J., Grantham-Hill, S., and Soto, D. (2015). On the independence of visual awareness and metacognition: a signal detection theoretic analysis. J Exp Psychol Hum Percept Perform *41*, 269-276.

S30. Yuval-Greenberg, S., Merriam, E.P., and Heeger, D.J. (2014). Spontaneous microsaccades reflect shifts in covert attention. J Neurosci *34*, 13693-13700.

S31. Duc, A.H., Bays, P., and Husain, M. (2008). Eye movements as a probe of attention. Prog Brain Res *171*, 403-411.
